# Starvation, Together with the SOS Response, Mediates High Biofilm-Specific Tolerance to the Fluoroquinolone Ofloxacin

**DOI:** 10.1371/journal.pgen.1003144

**Published:** 2013-01-03

**Authors:** Steve P. Bernier, David Lebeaux, Alicia S. DeFrancesco, Amandine Valomon, Guillaume Soubigou, Jean-Yves Coppée, Jean-Marc Ghigo, Christophe Beloin

**Affiliations:** 1 Institut Pasteur, Unité de Génétique des Biofilms, Paris, France; 2 Institut Pasteur, Génopole, Plate-forme 2–Transcriptome et Epigénome, Paris, France; University of Geneva Medical School, Switzerland

## Abstract

High levels of antibiotic tolerance are a hallmark of bacterial biofilms. In contrast to well-characterized inherited antibiotic resistance, molecular mechanisms leading to reversible and transient antibiotic tolerance displayed by biofilm bacteria are still poorly understood. The physiological heterogeneity of biofilms influences the formation of transient specialized subpopulations that may be more tolerant to antibiotics. In this study, we used random transposon mutagenesis to identify biofilm-specific tolerant mutants normally exhibited by subpopulations located in specialized niches of heterogeneous biofilms. Using *Escherichia coli* as a model organism, we demonstrated, through identification of amino acid auxotroph mutants, that starved biofilms exhibited significantly greater tolerance towards fluoroquinolone ofloxacin than their planktonic counterparts. We demonstrated that the biofilm-associated tolerance to ofloxacin was fully dependent on a functional SOS response upon starvation to both amino acids and carbon source and partially dependent on the stringent response upon leucine starvation. However, the biofilm-specific ofloxacin increased tolerance did not involve any of the SOS-induced toxin–antitoxin systems previously associated with formation of highly tolerant persisters. We further demonstrated that ofloxacin tolerance was induced as a function of biofilm age, which was dependent on the SOS response. Our results therefore show that the SOS stress response induced in heterogeneous and nutrient-deprived biofilm microenvironments is a molecular mechanism leading to biofilm-specific high tolerance to the fluoroquinolone ofloxacin.

## Author Summary

Biofilm surface-attached communities have the capacity to tolerate high concentrations of antibiotics, and bacterial biofilms formed on indwelling medical devices are difficult to eradicate and often lead to the onset of chronic or systemic infections. The physiological heterogeneity of multicellular biofilms has been associated with development of subpopulations highly tolerant to multiple antibiotics. Here we demonstrate that, upon starvation for specific essential growth nutrients, biofilm bacteria become highly tolerant to fluoroquinolone ofloxacin. The SOS response plays a critical role in this phenomenon, while the stringent response plays only a minor role. Taken together, these results support the hypothesis that bacteria localized within nutrient-limited niches of the biofilm structure may temporarily enter a physiological state enabling them to tolerate bactericidal concentrations of antibiotics.

## Introduction

Formation of bacterial biofilms on medical implants is a major health threat due to their high levels of tolerance to multiple antibiotics [1]. Biofilm-associated antibiotic tolerance is mainly attributed to two distinct processes: persistence and drug indifference, which both characteristically disappear once multicellular conditions subside [2], [3]. Persistence occurs in subpopulations of slow or non-growing bacteria, whereas drug indifference is exhibited by the entire population [4], [5]. Although the molecular bases of persistence are under active investigation [5], [6], drug indifference is far less well understood and is hypothesized to be multifactorial and to result from reduced antibiotic diffusion to slow growth rate of many cells within biofilms [7]. Alternatively, local gradients of nutrients, oxygen, pH, signalling molecules and waste products, as well as genetic heterogeneity, which can arise through mutations, recombination, and stochastic gene expression, could lead to physiological adaptation and drug indifference in heterogeneous biofilms [7]–[12]. Biofilm heterogeneity creates specialized niches in which bacteria respond to local cues, leading to genetically and metabolically distinct subpopulations exhibiting high tolerance to extracellular stresses such as antibiotics.

Since physical isolation of biofilm subpopulations is technically challenging [13], [14], analyses of antibiotic tolerance have thus far relied mainly on isolation of mutants with decreased ability to form tolerant biofilms. However, biofilm heterogeneity and physiologically specialized subpopulations limit the power of these strategies. As an alternative approach to investigating the mechanisms of biofilm-associated tolerance to antibiotics, we mutagenized a biofilm-forming *Escherichia coli* strain to identify mutants forming biofilms with increased tolerance towards two bactericidal antibiotics, ticarcillin, a ß-lactam-targeting peptidoglycan and ofloxacin, a fluoroquinolone targeting DNA gyrase. We reasoned that mutants forming highly antibiotic tolerant biofilms could correspond to mutations causing all biofilm bacteria to adopt physiological states usually only transiently expressed by biofilm subpopulations.

In this study, we found that biofilms formed by amino acid auxotroph mutants display high antibiotic tolerance and we demonstrated that amino acid starvation strongly increases the antibiotic tolerance of biofilm bacteria. As expected, tolerance to the ß-lactam ticarcillin correlated with slow growth displayed by all tested amino acid auxotrophs. However, we demonstrated that starvation for most amino acids induced tolerance towards ofloxacin only under biofilm conditions. While this biofilm-specific tolerance was shown to depend partially on a functional stringent response in leucine-starved biofilms, we demonstrated that carbon source starvation also mediates ofloxacin tolerance in biofilm and that the SOS response appears to be necessary for the ofloxacin tolerance exhibited upon both amino acid and carbon source starvation. However, we showed that SOS-dependent biofilm-specific ofloxacin tolerance is independent of toxin-antitoxin systems induced by the SOS response and previously associated with bacterial persistence. Consistently, we observed that SOS-dependent ofloxacin tolerance increases with biofilm age. Since local nutrient deprivation is a characteristic of aging and heterogeneous biofilms, our study therefore reveals a general mechanism linking nutritional stress and reversible biofilm-specific tolerance to the fluoroquinolone ofloxacin.

## Results

### An *in vitro* model for studying antibiotic tolerance in *E. coli* biofilms

**Figure 1:**
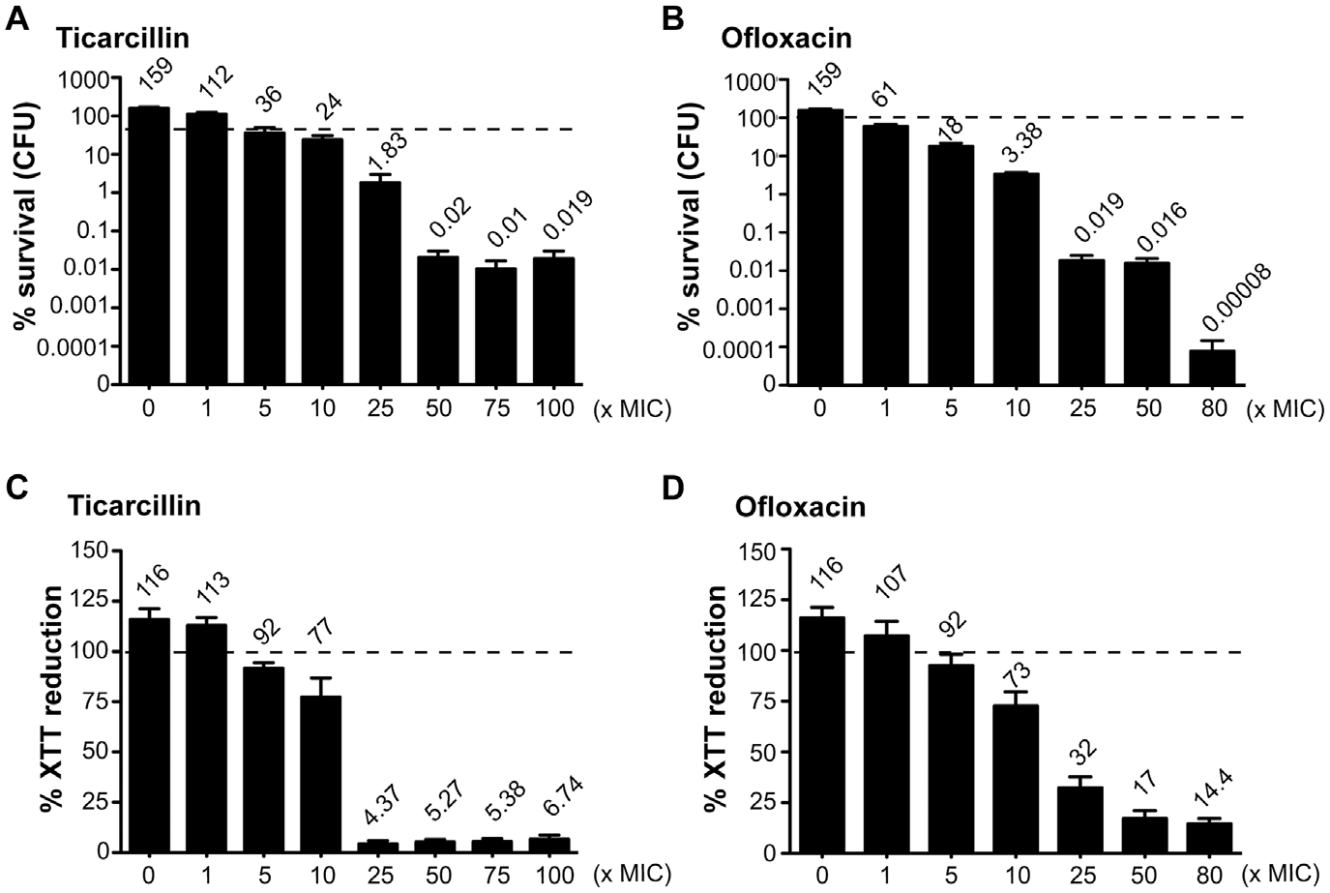
Antibiotic-tolerant populations of *E. coli* K-12 TG1 biofilms. Twenty-four-hour biofilms of *E. coli* TG1 were preformed in M63B1_Gluc_ and treated for a period of 24 h with concentrations up to 100- and 80-times the MIC values for ticarcillin and ofloxacin, respectively. Untreated controls represent 48 h biofilms in which fresh M63B1_Gluc_ without antibiotic was added after 24 h, explaining their values above 100%, represented by a dotted line in each panel. Viable cells of the treated biofilm population were quantified by viable cell counts (A and B) and the XTT-reduction assay (C and D) and were compared to numbers obtained prior to antibiotic treatment. % survival (CFU) values are indicated on top of each bar and are means ± standard error of the means (SEM) of at least six replicates.

In order to study antibiotic tolerance in biofilms, we grew static biofilms in M63B1_Gluc_ minimal medium in 96-well PVC microtiter plates using TG1, a previously described highly adherent *E. coli* K-12 strain expressing the F-conjugative pilus, involved in both conjugation and biofilm formation [15]. Twenty-four-hour *E. coli* TG1 biofilms (Figure S1) were treated with either ticarcillin, a β-lactam carboxypenicillin with bactericidal activity only against rapidly growing cells [16], or ofloxacin, a fluoroquinolone active against both growing and non-growing cells [17], [18]. We determined the viability of antibiotic-treated TG1 biofilm bacteria using both viable cells counts (CFUs) and bacterial metabolic activity (XTT-reduction assay) as survival read-outs. Although the antibiotic-tolerant population appeared greater when quantified using the XTT-reduction assay compared to the CFU count, both methods gave similar survival profiles in 24 h *E. coli* TG1 biofilms exposed to up to 80- and 100-times the MIC values for ofloxacin and ticarcillin, respectively (Figure 1A–1D). These surviving bacteria, which tolerated either ticarcillin (100× MIC) or ofloxacin (80× MIC), did not correspond to antibiotic-resistant mutants, since they displayed wild-type resistance profiles once re-inoculated and grown under classical planktonic conditions (data not shown). Similar results were obtained using a different biofilm-forming *E. coli* K-12 strain constitutively expressing type 1 fimbriae (data not shown), therefore demonstrating that biofilm formation *per se*, rather than the nature of the surface adhesin promoting biofilm formation, is involved in antibiotic tolerance of *E. coli* K-12 biofilms in our model.

### Amino acid auxotroph mutants exhibit increased antibiotic tolerance in biofilms

To identify mutations leading to high antibiotic tolerance transiently displayed by *E. coli* biofilm bacteria, we performed random *mariner* transposon mutagenesis on *E. coli* TG1 derivative strain TG1*gfp*. In our *in vitro* biofilm model, we screened approximately 10,000 mutants for homogeneous increased tolerance to ticarcillin (100× MIC) or ofloxacin (50× MIC) using the XTT-reduction assay as a high throughput survival read-out. We identified a total of 18 transposon mutants with increased biofilm tolerance to these two antibiotics when compared to their parental strain, and 16 of these mutants displayed auxotrophy to various amino acids (Table 1). Transposon mapping performed on 10 of these auxotrophic mutants indeed showed mutations in genes involved in amino acid biosynthesis, including four insertions in *leuC* and two in *proA*, indicative of saturated mutagenesis (Table 1). These 10 mapped mutants displayed amino acid auxotrophies to leucine (*leuB*; *leuC*), proline (*proA*), arginine (*argE*), isoleucine/valine (*ilvC*) and aromatic amino acids (*aroE*) when grown on minimal medium containing glucose as sole carbon source (data not shown). In addition, we determined that six of the remaining unmapped mutants also displayed auxotrophies to five different amino acids (proline, threonine, histidine, cysteine, and tyrosine). Our screen also identified two prototrophic transposon mutants (*rseC* and *pnp*) displaying slight increased tolerance in biofilms, which were not further investigated in this study.

**Table 1:** Auxotrophic transposon mutants with increased antibiotic tolerance in biofilms.

Gene-transposon insertion *	Auxotrophy type	Mutant ID
*leuC*	Leucine	36B6
*leuC*	Leucine	39D8
*leuC*	Leucine	91G7
*leuC*	Leucine	104H7
*leuB*	Leucine	50D6
*ilvC*	Isoleucine and valine	47E6
*aroE*	Aromatic amino acids**	44F5
*argE*	Arginine	70C4
*proA*	Proline	79E4
*proA*	Proline	94H3
N.D.	Cysteine	84A12
N.D.	Histidine	57A6
N.D.	Proline	3H4
N.D.	Threonine	17E11
N.D.	Threonine	84C10
N.D.	Tyrosine	102A7

* Transposon inserted at different locations within *leuC* and *proA*.

** Phenylalanine, tryptophan and tyrosine.

N.D. Not determined.

Formation of antibiotic-tolerant biofilms in M63B1_Gluc_ minimal medium by amino acid auxotrophs was intriguing, and suggested that use of overnight LB culture inoculum provided enough amino acids to support initial growth and biofilm development (data not shown). Indeed, we determined that supplementation of M63B1_Gluc_ with as little as 25 µg ml^−1^ of the required amino acid was sufficient to promote growth and formation of antibiotic-tolerant biofilm by all tested *E. coli* TG1 auxotrophs when treated in amino-acid-free medium (M63B1_Gluc_) (see Materials and Methods and data not shown).

### Starvation leads to increased antibiotic tolerance in biofilms

The identification, via our mutagenesis screen, of mutants auxotrophic for at least 12 amino acids suggested a link between biofilm antibiotic tolerance and amino acid starvation (Table 1). To confirm this relationship, we created *E. coli* TG1 amino acid auxotrophs that resulted from deletion of a single amino acid biosynthesis gene, including leucine, isoleucine, histidine, arginine, cysteine, methionine, lysine, proline, phenylalanine, tyrosine, tryptophan, glutamic acid, glycine, glutamine, serine and threonine (Table S1). However, auxotrophies for aspartic acid, asparagine, and alanine were not constructed due to the existence of multiple corresponding metabolic pathways. Similarly, we did not investigate auxotrophy for valine, since such mutants cannot be obtained without affecting isoleucine biosynthesis.

Among the 16 newly constructed amino acid auxotrophs, auxotrophy for glutamic acid, glycine, glutamine, serine and threonine showed reduced biofilm formation compared to the parent prototroph even when supplemented with 25 µg ml^−1^ of the required amino acid, and thus they could not be meaningfully tested for biofilm antibiotic tolerance (data not shown). Eleven of them, however, formed wild-type biofilms when supplemented with 25 µg ml^−1^ of the required amino acid (data not shown).

**Figure 2:**
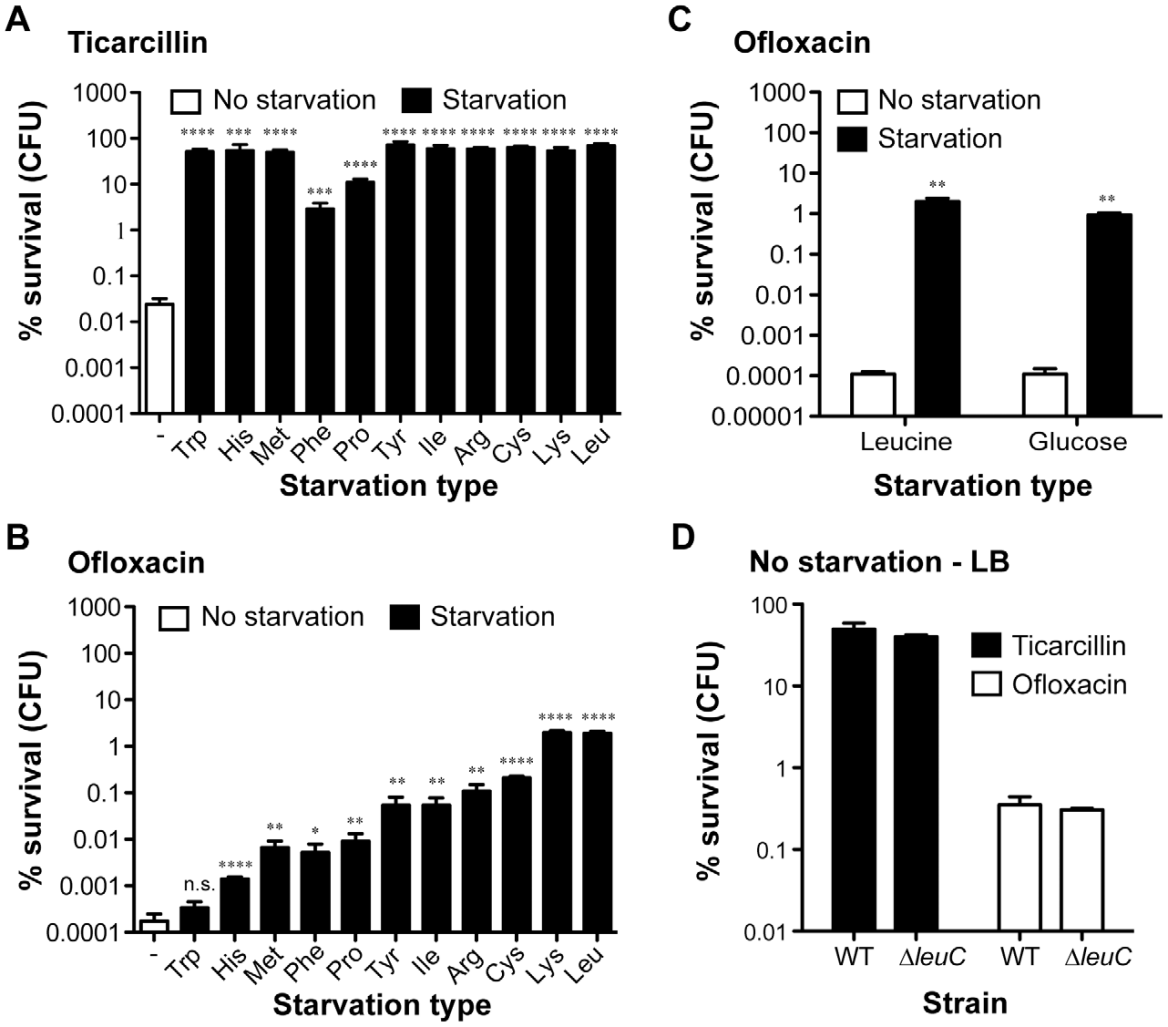
Starvation leads to high antibiotic tolerance in biofilms of *E. coli* TG1. The tolerance of non-starved biofilms (white bars; wild-type prototroph TG1 (WT)) to (A) ticarcillin (100 µg ml^−1^, 100× MIC) or (B) ofloxacin (5 µg ml^−1^, 80× MIC) was compared to biofilms starved for individual amino acids (black bars; auxotrophic mutants). (C) Addition of exogenous leucine (10 µg ml^−1^) or glucose (0.4%) restored ofloxacin sensitivity to biofilms. (D) Antibiotic tolerance profiles of biofilms grown and treated in LB-rich medium were indistinguishable from a leucine auxotroph and its WT prototroph. For panels A to C, biofilms of all tested amino acid auxotrophs were grown in M63B1_Gluc_ for 24 h with the addition of 25 µg ml^−1^ of the required amino acid, while WT was grown in M63B1_Gluc_ only. Antibiotic treatments were performed for a period of 24 h on 24-h biofilms. Conditions of starvation during treatment were achieved by removing the required amino acids (M63B1_Gluc_) for the respective auxotrophic strains or glucose for the WT prototroph (M63B1). In panel D, biofilms were grown for 24 h and treated for 24 h in LB-rich medium. Surviving cells were quantified by viable cell counts. Percent survival represents viable cells after 24 h of treatment compared to untreated biofilm prior to addition of antibiotics. All compared biofilms had similar numbers of CFU prior to antibiotic treatment (data not shown). Data represented are means ± SEM of at least three replicates. Asterisks indicate values significantly different from conditions of no starvation by the two-tailed unpaired t test: * *P*≤0.05, ** *P*≤0.01, *** *P*≤0.001, **** *P*≤0.001 and n.s. (not significant). The genotypes of all amino acid auxotrophic mutants constructed in the WT prototroph TG1 genetic background are described in Table S1 or as follows: tryptophan (Trp; Δ*trpA*::KmFRT), histidine (His; Δ*hisG*::KmFRT), methionine (Met; Δ*metA*::KmFRT), phenylalanine (Phe: Δ*pheA*::KmFRT), proline (Pro: Δ*proC*::KmFRT), tyrosine (Tyr; Δ*tyrA*::KmFRT), isoleucine (Ile; Δ*ilvA*::KmFRT), arginine (Arg; Δ*argH*::KmFRT), cysteine (Cys; Δ*cysD*::KmFRT), lysine (Lys; Δ*lysA*::KmFRT), and leucine (Leu: Δ*leuC*::GB).

To evaluate the impact of starvation of a single amino acid upon the antibiotic tolerance of biofilm bacteria, we compared survival of starved auxotrophic biofilms formed by these 11 auxotrophs to that of the wild-type prototroph parental strain TG1. During the starvation process, bacterial growth was blocked due to the use of auxotrophic mutant strains in the absence of the required amino acid. In these experimental conditions, we observed that biofilm formed by histidine, methionine, phenylalanine, proline, tyrosine, isoleucine, arginine, cysteine, lysine and leucine auxotrophs survived significantly better than prototrophic TG1 biofilms exposed to either ticarcillin (100× MIC) or ofloxacin (80× MIC) (Figure 2A and B; *P*<0.05). In the case of the tryptophan auxotroph, increased survival was observed upon exposition to ticarcillin only (Figure 2A) indicating that growth arrest was not the only mechanism involved in ofloxacin tolerance upon starvation to the other amino acids (Figure 2B). The tolerance levels exhibited by starved biofilms (individual amino acid) were greater towards ticarcillin than ofloxacin confirming that fluoroquinolone antibiotics like ofloxacin kill bacterial cells independently of growth.

To further investigate the contribution of starvation to antibiotic tolerance in biofilms, we chose auxotrophy for leucine as a model of auxotrophy-induced tolerance. We observed that blocking starvation in leucine auxotroph biofilms by exogenous addition of leucine (5–10 µg ml^−1^) reverted both ofloxacin and ticarcillin tolerance to levels observed in non-starved wild-type TG1 biofilms (Figure 2C and Figure S2). To determine whether exposure to other nutritional stresses could lead to a similar antibiotic tolerance profile in starved prototroph biofilms, we exposed biofilms formed by wild-type TG1 to antibiotics in glucose-free medium (M63B1- no carbon source). Under these conditions, we observed that deprivation of all carbon sources (glucose), and therefore absence of growth, increased the antibiotic tolerance of TG1 biofilms towards both ofloxacin and ticarcillin (Figure 2C and data not shown). Consistently, leucine auxotroph biofilms grown and treated in rich LB medium displayed wild-type sensitivity to both ticarcillin and ofloxacin, thereby demonstrating that amino acid shortage *per se* is involved in biofilm-associated antibiotic tolerance (Figure 2D). Biofilms grown in LB rich medium were more tolerant than those grown in minimal medium (M63B1_Gluc_) for both ofloxacin and ticarcillin (Figure 2A, 2C and 2D) suggesting the existence, in this rich medium, of other antibiotic tolerance mechanisms such as those mediated by indole production [19].

Altogether, these results demonstrated that starvation for essential growth nutrients such as amino acids and carbon sources increased the tolerance of *E. coli* K-12 biofilms to ticarcillin and ofloxacin. Further, we demonstrate that the use of auxotrophic mutant strains is an effective genetic strategy to adequately control and measure the impact of a specific starvation type such as a single amino acid.

### Ofloxacin hypertolerance upon starvation is biofilm-specific

**Figure 3:**
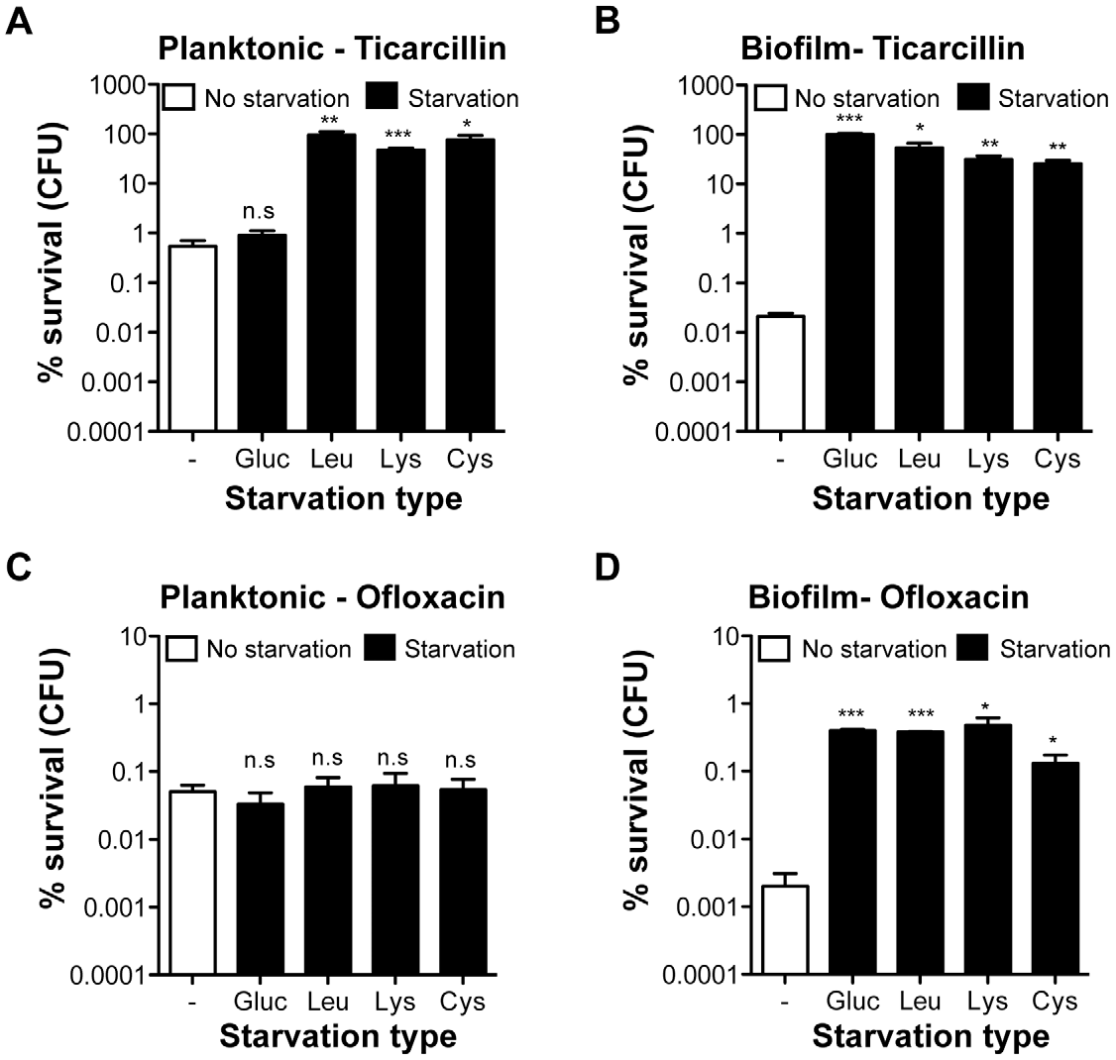
Antibiotic tolerance profile of starved planktonic and biofilm bacteria. Static cultures were grown for 24 h in M63B1_Gluc_ for wild-type prototroph TG1 (WT) and with the addition of 25 µg ml^−1^ of leucine, lysine, or cysteine for the corresponding auxotrophs. Planktonic bacteria from the 24-h static culture were removed from each well and therefore separated from the attached-biofilm cells. (A, C) Planktonic bacteria were then collected, spun, washed and treated in parallel with the biofilm bacteria (B, D) with ticarcillin (100 µg ml^−1^, 100× MIC) or ofloxacin (5 µg ml^−1^, 80× MIC) in the absence of glucose for WT prototroph TG1 (M63B1 medium) or amino acids for the different auxotrophic mutant strains (M63B1_Gluc_ medium) for 24 h. Survivor cells were quantified by viable cell counts. Percent survival represents the tolerant population after 24 h of treatment compared to the total number of CFU prior to addition of antibiotics. Equivalent CFU were present in all compared planktonic populations before antibiotic treatment. Data represented are means ± SEM of at least three replicates. Asterisks indicate values significantly different from no starvation condition by the two-tailed unpaired t test: * *P*≤0.01, ** *P*≤0.001, *** *P*≤0.0001 and n.s. (not significant). The genotypes of the three amino acid auxotrophic mutants constructed in the WT prototroph TG1 genetic background are described in Table S1 or as follows: leucine (Leu: Δ*leuC*::GB), lysine (Lys; Δ*lysA*::KmFRT), and cysteine (Cys; Δ*cysD*::KmFRT).

To determine whether increased antibiotic tolerance of starved amino acid auxotrophs was specific to the biofilm lifestyle, we compared the tolerance profiles of planktonic and biofilm bacteria upon starvation to glucose, leucine, lysine, and cysteine (Figure 3). To compare planktonic and biofilm lifestyles, planktonic cells were harvested from the same wells in which tolerant biofilms were formed, thereby growing both cell types under identical conditions. Interestingly, planktonic bacteria were more tolerant than biofilm bacteria to both ticarcillin and ofloxacin in non-starving conditions. In starvation conditions, with the exception of glucose, free-swimming planktonic bacteria harvested from the three chosen auxotrophs continued to display increased tolerance to ticarcillin like their biofilm counterparts (Figure 3A and 3B). In contrast with observations made under biofilm conditions (Figure 2B and Figure 3D), planktonic cells starving for glucose, leucine, lysine or cysteine did not exhibit increased tolerance to ofloxacin (Figure 3C). These results therefore indicated that, although ticarcillin tolerance displayed by starved bacteria in planktonic and biofilm conditions is likely a consequence of reduced growth or its absence, the increased tolerance to ofloxacin displayed by starved bacteria is biofilm-specific.

### The biofilm-specific ofloxacin hypertolerance partially relies on the stringent response upon leucine starvation

The biofilm specificity of ofloxacin hypertolerance led us to speculate that a mechanism other than growth arrest could be involved in generating highly tolerant populations when starved of specific nutrients. Bacteria have evolved various general response mechanisms towards extracellular stresses with the potential of promoting antibiotic tolerance [20]. Starvation induces the so-called stringent response that is characterized by the synthesis of (p)ppGpp by both RelA and SpoT [21], which was recently linked to antibiotic tolerance in nutrient-limited biofilms [12]. The potential involvement of the stringent response in the biofilm-specific ofloxacin hypertolerance was first assayed by measuring the tolerance of Δ*relA* in both wild-type TG1 and its derivative leucine auxotroph upon starvation for glucose (Figure S3A) or leucine (Figure S3B), respectively. The impairment of the stringent response by Δ*relA* reduced the ofloxacin tolerance normally exhibited by biofilms starved for leucine without totally abolishing it (Figure S3B). As expected, since starvation to carbon sources like glucose is under the control of SpoT [22], the high ofloxacin tolerance exhibited upon glucose starvation was still displayed by Δ*relA* biofilms supporting the absence of role for RelA upon glucose starvation (Figure S3A). Unfortunately, it was impossible to meaningfully determine the role of SpoT on the biofilms ofloxacin tolerance upon glucose starvation with our experimental conditions. Indeed, in *E. coli* K-12, a Δ*spoT* mutation is lethal in a wild-type background of *E. coli* K-12 and a (p)ppGpp0 strain (Δ*relA*Δ*spoT*) is itself auxotrophic to multiple amino acids [23].

These partial results, mainly due to genetic incompatibilities, at least suggest that stringent response through RelA partially contributes to ofloxacin hypertolerance exhibited by *E. coli* K-12 biofilms upon leucine starvation.

### The SOS stress response is fully required for the starvation-induced biofilm ofloxacin tolerance

Although a Δ*relA* mutation partially restored wild-type sensitivity to ofloxacin upon leucine starvation, their biofilms were still more tolerant than non-starved wild-type biofilms (Figure S3B). To determine whether a parallel mechanism was also involved in the biofilm-specific ofloxacin tolerance, we decided to focus our attention on the SOS response. The SOS response is known for its role in DNA repair and mutagenesis, however it was also shown to influence the formation of planktonic *E. coli* cells persistent to fluoroquinolone antibiotics [24]–[26]. Once induced, the SOS response is triggered upon RecA-dependent cleavage of LexA, the repressor of SOS response-regulated genes [27].

**Figure 4:**
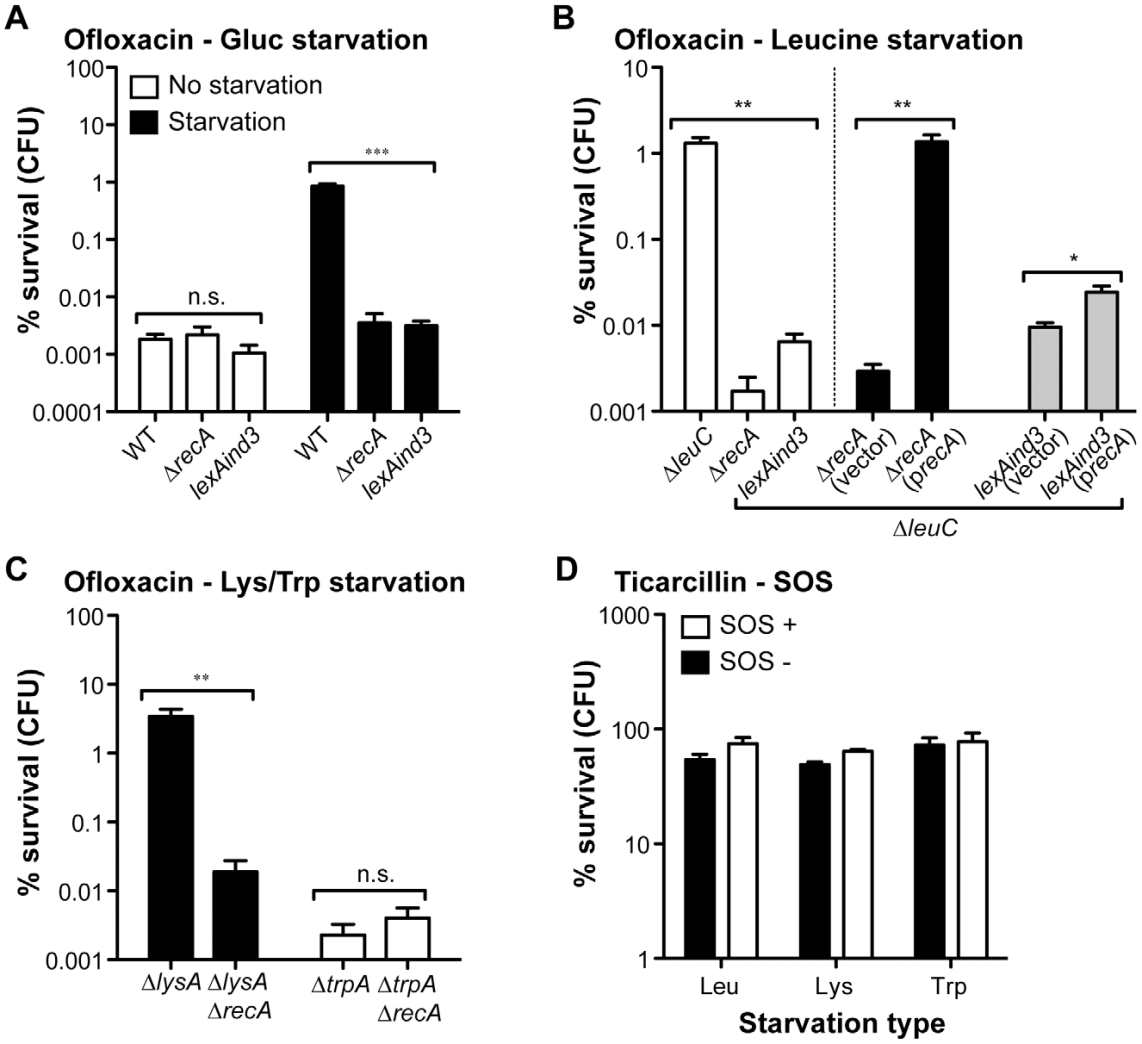
The high ofloxacin tolerance exhibited by starved biofilms is SOS-response-dependent. The impact of SOS response loss-of-function mutations Δ*recA* and *lexAind3* on the ofloxacin tolerance of starved biofilms for glucose (A), leucine (B), lysine and tryptophan (C). Briefly, all biofilms were grown for 24 h in M63B1_Gluc_ for all prototrophic strains and with the addition of 25 µg ml^−1^ of leucine, lysine or tryptophan for corresponding auxotrophic strains. All biofilms were treated for 24 h in M63B1_Gluc_ containing ofloxacin (5 µg ml^−1^). For the glucose starvation environment, M63B1 without glucose was used instead of M63B1_Gluc_ (Panel A - black bars). Ofloxacin hypertolerance of *ΔleuCΔrecA* was not restored when adding the vector control plasmid pAM34 (vector), but complete restoration of high ofloxacin tolerance upon leucine starvation was observed by *in trans* complementation using pAM34*recA* (p*recA*). Complementation of Δ*leuClexAind3* with pAM34*recA* (p*recA*) failed to fully restore ofloxacin hypertolerance back to leucine auxotroph levels. Both pAM34 and pAM34*recA* were maintained by the presence of 0.5 mM IPTG. (D) The SOS response mutation Δ*recA* (SOS-) did not affect ticarcillin hypertolerance of starved biofilms for leucine (Leu - *ΔleuCΔrecA*), lysine (Lys – *ΔlysAΔrecA*) or tryptophan (Trp - *ΔtrpAΔrecA*). Viable cells of the treated biofilm population were quantified by viable cell counts. Percent survival represents the tolerant population after 24 h of treatment compared to untreated biofilm prior to addition of antibiotics. All compared biofilms had similar numbers of CFUs prior to antibiotic treatment (data not shown). Data represented are means ± SEM of at least three replicates. Asterisks indicate values significantly different by the two-tailed unpaired t test: * *P*≤0.05, ** *P*≤0.01, *** *P*≤0.001, and n.s. (not significant). All strains used here were originally constructed in the WT TG1 genetic background and are detailed in Table S1. The different SOS response mutant strains made in amino acid auxotrophic background are derivatives of Δ*leuC*::ΔFRT (Leu - leucine), Δ*lysA*::ΔFRT (Lys - lysine), and Δ*trpA*::ΔFRT (Trp – tryptophan).

To test the potential contribution of the SOS response to hypertolerance of biofilms starved for ofloxacin, we introduced a Δ*recA* into selected auxotrophs (leucine, lysine, and tryptophan) and a *lexAind3* mutation encoding an uncleavable LexA protein [28] into a leucine auxotroph. These mutations were also introduced into their prototrophic parental strain TG1 (Table S1). We first determined that these two loss-of-function mutations had no significant effect on ofloxacin tolerance displayed by non-starved wild-type *E. coli* TG1 biofilms (Figure 4A). We then observed that biofilm-associated ofloxacin tolerance of Δ*recA* biofilm bacteria starved for glucose, leucine and lysine was reduced to levels comparable to those of non-starved biofilms (Figure 4A, 4B, and 4C). Moreover, ofloxacin hypertolerance was also reduced for *lexAind3* biofilms starved for glucose or leucine (Figure 4A and 4B). In contrast, the ofloxacin tolerance of biofilms starved for tryptophan was not affected by a Δ*recA* mutation, consistent with our previous observations showing that tryptophan starvation did not affect ofloxacin tolerance in biofilms (see Figure 2B). Consistently, biofilm-associated ofloxacin tolerance was fully restored to levels observed upon leucine starvation after introduction of a wild-type copy of *recA* in a leucine auxotroph background (Figure 4B). We also showed that overexpression of RecA in a *ΔleuClexAind3* background failed to restore ofloxacin hypertolerance in biofilms formed by the leucine auxotroph, therefore demonstrating that this phenotype is SOS-response-dependent and not solely RecA-dependent (Figure 4B). Furthermore, a Δ*recA* mutation had no effect on ticarcillin tolerance of biofilms starved for leucine, lysine or tryptophan (Figure 4D) suggesting that growth arrest is the likely mechanism involved in ticarcillin resistance upon starvation.

We showed that the induced ofloxacin tolerance upon starvation was unique to the biofilm stage (Figure 3C and 3D). This was also confirmed by demonstrating that the SOS response, critical for biofilm bacteria (Figure 4), had no significant role in the ofloxacin tolerance of starved planktonic cells (Figure S4). Our genetic-based results confirmed that a functional SOS response system was fully required to tolerate high levels of ofloxacin for biofilm bacteria starving for glucose, leucine, or lysine.

The SOS-dependence of starved biofilms ofloxacin tolerance could potentially be due to an induction of the SOS response either during biofilm formation and/or by the ofloxacin treatment. In fact, fluoroquinolone antibiotics such as ofloxacin are known to induce the SOS response at subinhibitory and bactericidal concentrations, however, in conditions where bacteria exhibit growth [24], [29]. The ofloxacin treatment used in our experiments was performed in starvation condition, i.e. in absence of growth for the auxotrophic mutants or the prototrophic WT treated without glucose. Therefore, our current results would favour the idea that the SOS response cannot be induced during the ofloxacin treatment mainly because of a complete shutdown of both transcription and protein translation and thus pointing towards a role of the SOS response in ofloxacin tolerance during the process of biofilm formation itself. To provide evidence of this shut down of protein translation during starvation, here we used an *E. coli* strain containing a *lacZ* gene under the control of the P_LtetO-1_ promoter inducible by anhydrotetracycline (aTc). We showed that a 24-h biofilm of this strain subjected to glucose starvation was unable to translate *lacZ* in presence of aTc unlike unstarved 24-h biofilm (Figure S5).

Taken together, this strongly suggests that ofloxacin tolerance of biofilm is linked to the presence of a functional SOS response during biofilm formation and is only visible in condition where biofilms are subjected to a strong nutritional stress.

### SOS-dependent toxin-antitoxin modules do not influence the ofloxacin hypertolerance of starved biofilms

The SOS system of *E. coli* involves the induction of at least 40 genes, for which the expression of specific toxin-antitoxin (TA) modules was previously associated with the SOS response, antibiotic tolerance and amino acid starvation [5], [25], [30]. To investigate whether TA modules that are part of the SOS regulon were directly responsible for the described biofilm-associated ofloxacin tolerance upon starvation, we first deleted the genes encoding the four currently known functional SOS-dependent TA modules TisAB, SymER, DinJ/YafQ and YafNO (the HokE toxin is inactive in *E. coli* K-12 [31]) in the biofilm-forming background of MG1655Δ*leuC* carrying the F episome (F'Tet; Table S1). Simultaneous deletion of these four SOS-dependent TA modules did not affect the ofloxacin tolerance profile of leucine-starved biofilms (Figure S6A). Although not regulated by the SOS response, we also evaluated the role of other well-characterized TA modules [32] some of which have been shown to be involved in antibiotic bacterial persistence as well as the Lon protease known to regulate TA protein stability [33]. However, individual deletion of the gene coding for the toxin of non-SOS TA modules (RelE, HipA, MazF, ChpB, YoeB, HicA as well as CcdB, located on the F plasmid) and for the Lon protease in a leucine auxotroph background of strain TG1 did not reduce ofloxacin tolerance of leucine-starved biofilms (Figure S6B).

These results showed that, although a functional SOS stress response is critical for the increased tolerance to ofloxacin displayed by starved biofilms, this tolerance is exerted independently of known SOS induced TA modules and is maintained in strains carrying single deletion of the best-characterized SOS-independent TA modules.

### SOS-dependent biofilm tolerance to ofloxacin increases with biofilm age

**Figure 5:**
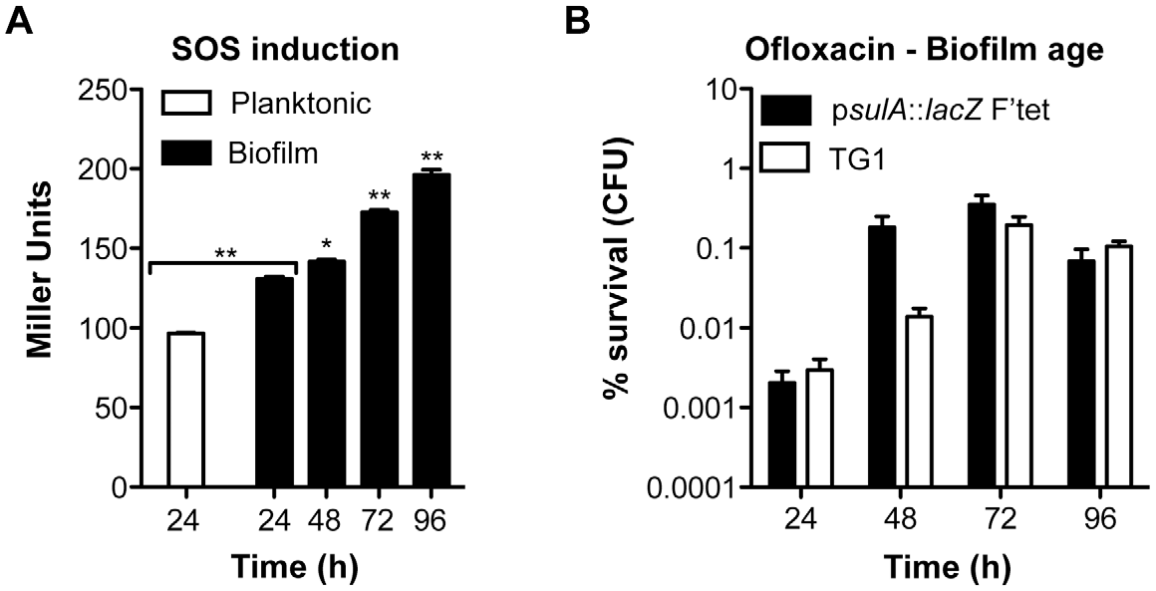
Induction of the SOS response and ofloxacin tolerance in aging biofilms. (A) Induction of the SOS response was monitored by β-galactosidase assay in aging biofilms and planktonic cells using an *E. coli* K-12 derivative carrying a p*sulA*::*lacZ* transcriptional fusion and the biofilm promoting factor F'tet. (B) The influence of biofilm age on ofloxacin tolerance was evaluated in biofilms of the reporter strain p*sulA*::*lacZ* F'tet (black bars) and wild-type TG1 (white bars). Briefly, all biofilms were grown for 24, 48, 72 and 96 h in M63B1_Gluc_ with medium renewal every 24 h. In (B) biofilms were treated for 24 h with ofloxacin (5 µg ml^−1^, 80× MIC) in M63B1_Gluc_, therefore in absence of starvation. Survivors were quantified by viable cell counts. Percent survival represents the tolerant population after 24 h of treatment compared to untreated biofilm prior to addition of antibiotics. Data represented are mean ± SEM of at least three replicates. Asterisks indicate values significantly different than 24-h biofilms. Statistics were performed by the two-tailed unpaired t-test: * *P*≤0.001 and ** *P*≤0.0001.

**Figure 6:**
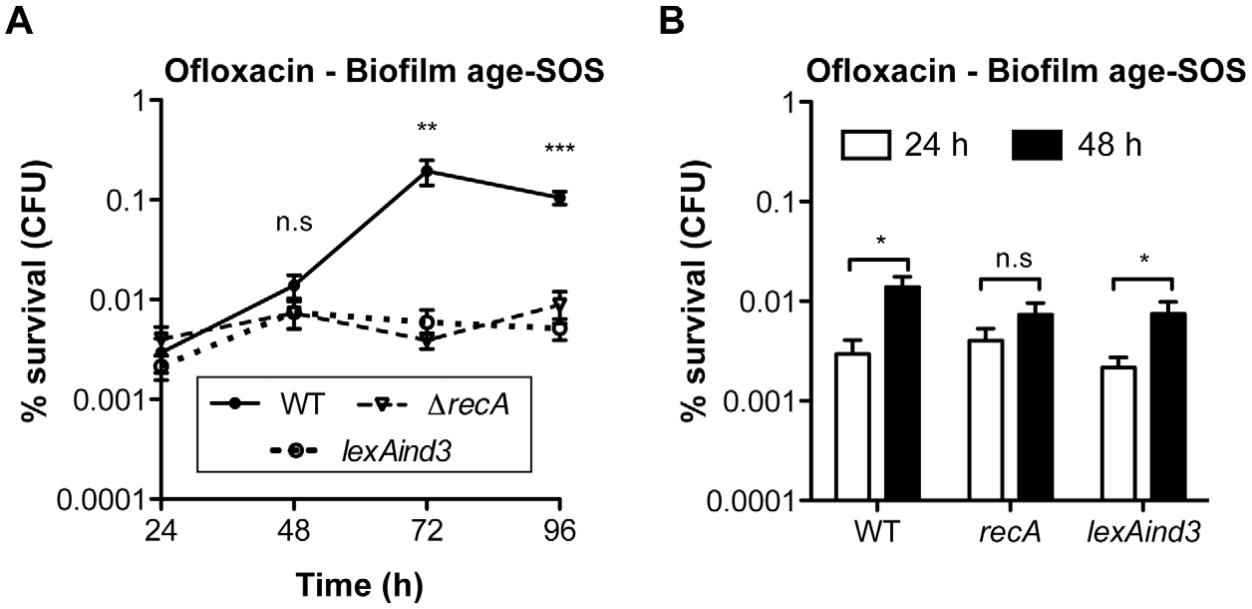
Ofloxacin tolerance in aging biofilms is SOS-response-dependent. (A) The influence of the SOS response on the ofloxacin tolerance of aging biofilms was evaluated and compared between biofilms of wild-type TG1 (WT), Δ*recA*, and *lexAind3*. (B) A close-up of the data obtained in panel A for the 24- and 48-h biofilms. Briefly, all biofilms were grown for 24, 48, 72 and 96 h in M63B1_Gluc_ with medium renewal every 24 h and treated for 24 h with ofloxacin (5 µg ml^−1^, 80× MIC) in M63B1_Gluc_ (no starvation). Survivors were quantified by viable cell counts. Percent survival represents the tolerant population after 24 h of treatment compared to untreated biofilm prior to addition of antibiotics. All compared biofilms had similar numbers of CFUs prior to antibiotic treatment (data not shown). Data represented are mean ± SEM of at least three replicates. Asterisks indicate values significantly different than WT (panel A). Statistics were performed by the two-tailed unpaired t-test: * *P*≤0.05, ** *P*≤0.01, *** *P*≤0.0001, and n.s. (not significant). The genotypes of the different strains used are detailed in Table S1 or as follows: WT (TG1), Δ*recA* (TG1Δ*recA*::KmFRT ), and *lexAind3* (TG1*lexAind3*).

Biofilm specificity and SOS-dependence of this increased ofloxacin tolerance led us to speculate about the possible links between these two phenomena. Unlike planktonic bacteria, biofilms are heterogeneous environments in which bacteria can experience various stresses such as local transient nutritional deprivation, oxidative stress, local pH, and oxygen tension modification [7], [9], some of which may induce the SOS response [20], [34]–[37] and then influence biofilm antibiotic tolerance. Nutrient gradients or waste accumulation together with bacterial physiological heterogeneity are supposed to increase during biofilm maturation [7] and therefore potentially impact ofloxacin tolerance over time upon induction of the SOS response. To assess this hypothesis, we used aging biofilms to determine whether the SOS response was indeed induced over time. We monitored the expression of the SOS-regulated gene *sulA* using a *lacZ* transcriptional reporter fusion in an *E. coli* K-12 strain harbouring the biofilm-promoting factor F'tet. Transcription of *sulA* increased progressively over time in aging biofilms (Figure 5A) similarly to the increased ofloxacin tolerance exhibited in both the p*sulA*::*lacZ* reporter strain and TG1 (Figure 5B). Although a little difference was observed in ofloxacin tolerance of 48-h biofilms between the two strains, the overall progressive tolerance was similar in both genetic backgrounds (Figure 5B). Therefore, the induction of the SOS response occurring in aging biofilms (Figure 5A) can confidently be extrapolated to strain TG1. Consistently, the overall increase ofloxacin tolerance demonstrated in aging biofilm of wild-type TG1 (Figure 5B) was abolished in both SOS response mutants Δ*recA* and *lexAind3* (Figure 6A). A significant increase of ofloxacin tolerance was, however, observed between 24- and 48-h biofilms of strain *lexAind3* but not in a *recA* mutant suggesting that unknown SOS-independent tolerance mechanisms could act during certain biofilm development steps.

Additionally, planktonic bacteria displayed a significant reduction of *sulA* expression compared to their 24-h biofilm counterparts (Figure 5A). This is in agreement with our previous observations demonstrating that, in planktonic bacteria, ofloxacin tolerance was not increased upon starvation (Figure 3C) and that the SOS response was not involved in the ofloxacin tolerance of planktonic bacteria independently of their starvation state (Figure S4), unlike biofilm bacteria (Figure 4). Taken together, these results demonstrated that the SOS response was induced in aging biofilms which increasing tolerance to fluoroquinolone ofloxacin is SOS-response-dependent.

## Discussion

Identification of antibiotic tolerance determinants that are unique to biofilms has proven to be an arduous task, due in part to the physiological heterogeneity of these bacterial communities [1], [5], [7]. Here we screened for *E. coli* mutants forming biofilms with increased antibiotic tolerance in order to reveal transient physiological states and genetic modifications as tolerance mechanisms potentially occurring within biofilm subpopulations.

This approach showed that most identified mutants were amino acid auxotrophs displaying strong tolerance to either ticarcillin or ofloxacin upon starvation. Biofilm heterogeneity through generation of amino acid auxotrophs had been previously observed. *Pseudomonas aeruginosa* amino acid auxotrophs are commonly isolated from sputa of cystic fibrosis patients and are generally more resistant to antibiotics than their prototrophic parental strains [9], [10], [38], [39]. In contrast to the planktonic cells and biofilm ticarcillin tolerance displayed by all tested starved amino acid auxotrophs, leucine, lysine, and cysteine auxotrophs exhibited increased tolerance to ofloxacin, which occurred only in starved biofilms. Since ofloxacin is active even against non-growing bacteria [17], [18], general growth arrest did not explain the observed increase in ofloxacin tolerance. Moreover, while bacteria starved for glucose, leucine, cysteine, and lysine were highly tolerant to ofloxacin in biofilms, tryptophan starvation had no significant effect and starvation for other amino acids led to intermediate levels of tolerance. While these results confirm that the observed tolerance was not due to growth arrest, but rather to an active mechanism, it also suggests that distinct starvation types may have different physiological consequences in biofilms, consistent with a recent study demonstrating that starvation for different amino acids resulted in variations in growth rates and ß-galactosidase activity [40].

We demonstrated that both a functional RecA and a cleavable LexA were essential for the ofloxacin tolerance phenotype exhibited by biofilms starved either for glucose, leucine or lysine using prototrophic and auxotrophic mutant strains. A RecA-mediated SOS response was not necessary for increased ticarcillin tolerance in biofilms suggesting growth arrest as tolerance determinant unlike ofloxacin. We previously showed that *recA* and other SOS response genes were significantly induced in mature biofilms compared to exponentially grown planktonic cells [41]. Consistently, our findings demonstrated that induction of the SOS response was significantly greater in biofilm bacteria than in their planktonic counterparts. This could account for the biofilm specificity of ofloxacin tolerance, in which RecA or SOS-regulated proteins reached a critical level that is not achieved in planktonic cells, which may explain the lack of ofloxacin tolerance induction in starved planktonic populations.

Interestingly, despite a lower level of SOS-response, non-starved planktonic bacteria were more tolerant than their biofilm counterparts to both ticarcillin and ofloxacin, which is in agreement with previous observations [3], [17]. The ticarcillin tolerance could be explained by the fact that planktonic stationary cells are under growth arrest and therefore highly tolerant to ticarcillin while biofilms are heterogeneous populations containing a mix of growing and non-growing bacterial cells. For ofloxacin, the hypothesis of slow or absence of growth cannot satisfactorily explain the higher planktonic cells tolerance since it kills independently of growth. Stationary phase cultures are known to generate more persister cells than exponentially growing cultures, even more than biofilm cells, therefore suggesting some level of planktonic and biofilm specificity involved in generation of persisters [17]. In absence of starvation, it is therefore possible to imagine that a higher level of persisters occurring in planktonic cells would explain their higher tolerance. Moreover, the ofloxacin tolerance of planktonic bacteria is likely due to a mechanism other than the SOS response since a Δ*recA* did not significantly impair the overall tolerance of both non-starving and starving populations. The latter results strengthen the notion that the induction of ofloxacin tolerance in starving biofilms is likely to involve mechanisms different than those currently described in planktonic cells [5], [42], [43].

Although our study evaluates ofloxacin only, fluoroquinolones are known to induce the SOS response in *E. coli* [24], [29]. Here we showed that induction of the SOS response upon exposure to bactericidal concentrations of ofloxacin (80× MIC) was unlikely to occur in absence of carbon source, i.e. in full deprivation state, due to the absence of protein synthesis. Hence, both induction of the SOS response during biofilm formation and nutrient depletion (carbon source or amino acids) are necessary but not sufficient to lead to the observed biofilm-specific high tolerance to ofloxacin.

The SOS response is involved in bacterial adaptive responses and horizontal gene transfer potentially leading to the onset of antibiotic resistance in a broad range of bacterial species [44]–[49]. The SOS response was also previously shown to induce persistent cells in planktonic populations during treatment with the fluoroquinolone ciprofloxacin [24], [25]. “Persisters” are phenotypic variants that can revert to wild-type antibiotic sensitivity [4], [5]. They are believed to be the end result of stochastic endogenous stress leading to growth arrest, resulting in the shutdown of bactericidal antibiotic targets and therefore the creation of multidrug-tolerant cells [5], [6]. Among possible targets, the SOS response was shown to induce expression of the TA module TisAB [25]. However, TisAB-dependent ciprofloxacin persisters were only observed in exponentially growing bacteria [25], consistent with the fact that none of the TA modules tested in our study was required for the biofilm-associated increased tolerance to ofloxacin, including known SOS-induced TA modules TisAB, SymER, DinJ-YafQ and YafNO. Our results therefore indicate that starvation in biofilm bacteria induces a SOS-dependent ofloxacin tolerance, but independently of TA loci induced by the SOS response. As raised above, the ability in generating persisters differs greatly between planktonic and biofilms cells [17], therefore it can be speculated that ofloxacin-tolerant persisters in biofilms could be physiologically distinct from their planktonic persister counterparts. Future work should concentrate precisely on identifying which SOS-gene(s), among the over 40 known SOS-regulated genes [50], [51], is required to support this biofilm increased tolerance towards ofloxacin.

Since we showed that starvation, but not auxotrophy *per se*, promotes the high ofloxacin tolerance observed within biofilms, our results demonstrate a general link between nutrient limitation and formation of highly antibiotic-tolerant populations. Starvation to amino acids or carbon sources induces the stringent response through the synthesis of (p)ppGpp via both RelA and SpoT [21]. Indeed, while we could not assess the role of SpoT since a *relA spoT* double mutant, i.e. a ppGpp0 strain, is auxotroph to multiple amino acids [23], we demonstrated that *relA* plays a role in observed ofloxacin biofilm-specific hypertolerance. However, the role of this response seemed less important than that of the SOS response, since the stringent response was only partially involved following leucine starvation. Nguyen *et al.* recently demonstrated that antibiotic tolerance exhibited in nutrient-limited biofilms of *P. aeruginosa* depended on the stringent response [12]. It can therefore be imagined that multiple pathways might play a significant role in the biofilm-associated ofloxacin tolerance of starved biofilms, with their role depending on conditions prevailing within biofilms. The manner in which these two distinct responses become integrated at the molecular level to promote high biofilm-specific ofloxacin tolerance remains unclear at the moment.

Nutrient deprivation might occur in biofilm populations located in micro-niches of heterogeneous biofilms, leading to higher tolerance to antibiotics [7]. Alternatively, adverse physicochemical parameters such as oxygen rarefaction prevailing within deep biofilm layers could slow down bacterial metabolism, thereby causing bacteria to enter a quasi-auxotrophic state even in the presence of sufficient available carbon sources. In support of this hypothesis, we showed that ofloxacin tolerance increased with biofilm age, suggesting that nutrient-depleted pockets or starved layers of cells may increase in number or size with biofilm maturation, or else be accompanied by a reduction in nutrient flow. Interestingly, Allison *et al.* recently showed that reactivation of the metabolism of *E. coli* persister cells restored antibiotic sensitivity both in planktonic and biofilms cells [52]. In parallel with our study, the addition of exogenous leucine during antibiotic treatment of leucine auxotroph biofilms restored sensitivity to levels greater than those of the parent wild-type prototroph treated without leucine, suggesting that the addition of amino acids may also impact overall antibiotic tolerance. While the study by Allison *et al.* did not specifically involve ofloxacin, it clearly suggests that nutrient depletion might play a central role in the ability of biofilm-associated bacteria to tolerate otherwise lethal concentrations of antibiotics. Although lethal concentrations of ofloxacin induce the SOS response, we show here that increased tolerance of aging biofilms is SOS-dependent and is correlated with increased SOS induction in biofilm bacteria. While this result is consistent with SOS induction in aging biofilm-like *E. coli* colonies on agar plates [37], it also suggests that biofilm areas in which local nutrient depletion and/or various stresses (pH drop, reduction of oxygen, etc) occur may cause SOS induction and favour high antibiotic tolerance and thus persistence.

In conclusion, we show that starvation potentially occurring locally in heterogeneous and diffusion-limited biofilm microenvironments leads to biofilm-specific SOS-dependent tolerance to the fluoroquinolone ofloxacin. Identification of the SOS response as a key molecular determinant in antibiotic tolerance in nutrient-limited biofilms reveals a possible general mechanism leading to high, but transient tolerance to a medically relevant class of antibiotics. It underlines the importance of the SOS response in the different bacterial mechanisms counteracting the effects of antibiotics, and it raises the possibility of using the SOS response as a target for reducing the emergence of biofilm tolerance to antibiotics in clinical settings [53]–[55].

## Materials and Methods

### Bacterial strains, plasmids, and growth conditions

Bacterial strains and plasmids used in this study are described in Table S1. All experiments were performed in 0.4% glucose M63B1 minimal medium (M63B1_Gluc_) or in lysogeny broth (LB) medium [56] at 37°C unless specified otherwise. Antibiotics were added when required at the following concentrations: kanamycin (Km), 50 µg ml^−1^; tetracycline (Tet), 15 µg ml^−1^; chloramphenicol (Cm), 25 µg ml^−1^; spectinomycin (Spec), 25 µg ml^−1^. Amino acids were added to M63B1_Gluc_ when required to a final concentration varying from 1 to 100 µg ml^−1^. All chemicals were purchased from Sigma-Aldrich (St. Louis, MO).

### Biofilm formation assay

A static biofilm formation assay was performed as previously described using 96-well polyvinyl chloride (PVC) microtiter plates (Falcon; Becton Dickinson Labware, Oxnard, CA) [57], [58]. *Prototrophic bacteria*: Overnight cultures grown in LB medium with required antibiotic(s) were diluted into M63B1_Gluc_ to an OD_600_ of 0.05 and used as inoculum. *Auxotrophic bacteria*: One ml of an overnight culture grown in LB medium was washed twice in M63B1 (to remove excess amino acids), normalized to an OD_600_ of 0.05 in M63B1_Gluc_ and supplemented with 25 µg ml^−1^ of the lacking amino acid corresponding to each evaluated auxotroph. Each microtiter well was then inoculated with 100 µl of the OD_600_ 0.05 inoculum, with a minimum of three wells per bacterial strain for each assay, and incubated at 37°C for 24, 48, 72 or 96 hours with renewal of growth medium every 24 h.

### Biofilm bacteria/biomass detection methods

#### Crystal violet staining

Biofilms attached to the sides of microtiter wells were thoroughly rinsed in water to remove unattached bacterial cells and stained with 125 µl of crystal violet (1% v/v) as previously described [57], [58]. Quantification of the stained biomass was achieved by adding 150 µl per well of a dissolving solution (ethanol (80%)/acetone (20%) (v/v)). Subsequently, dissolution of the bound crystal violet in each well was quantified by spectrometry at an absorbance of 595 nm.

#### Metabolic activity of biofilms in microtiter wells

Metabolic activity associated with biofilm cells was determined by the XTT (2,3-bis 2-methoxy-4-nitro-5-sulfo-phenyl-2H-tetra-zolium-5-carboxanilide) reduction assay as previously described [59]. Briefly, each well containing biofilms was washed once with 125 µl of phosphate-buffered saline (PBS: 10 mM sodium phosphate, pH 7.4, 0.9% NaCl) to remove unattached cells, and filled with 125 µl of a PBS solution containing 50 µg ml^−1^ XTT and 1 µM menadione. Plates were incubated at 37°C for 3.5 h in the dark, allowing metabolically active bacterial cells to reduce the XTT, which was then quantified colorimetrically by measuring absorbance at 492 nm.

#### Colony-forming units (CFU) of biofilms in microtiter wells

CFU of biofilms attached to the sides of microtiter wells were determined. Each well containing a biofilm was washed once with M63B1 to remove unattached cells and filled with 100 µl of the same medium enabling bacterial survival without growth. CFU determination was performed by serial dilutions on washed biofilms that were mechanically disrupted by pipetting.

### Minimal inhibitory concentration (MIC) determination

MIC values of ticarcillin (Ticarpen; GlaxoSmithKline, Marly-le-Roi, France) and ofloxacin (Sigma-Aldrich) were determined by macrodilutions in M63B1_Gluc_ as previously described [60]. MICs of ticarcillin and ofloxacin for TG1*gfp* were determined to be 1 and 0.0625 µg ml^−1^, respectively.

### Antibiotic susceptibility assays

#### Biofilm bacteria

Biofilms were formed for 24 h as described above. Unattached and planktonic bacteria were first removed from 24 h pre-formed biofilms and wells were then filled with 100 µl of M63B1 containing a specific antibiotic concentration, unless noted otherwise. After 24 h of incubation at 37°C, the antibiotic susceptibility of the treated biofilm population versus a 24 h biofilm was determined by CFU counts and/or colorimetric differences using the XTT-reduction assay as mentioned above.

#### Planktonic bacteria

Bacterial cultures were grown statically using 96-well microtiter plates for 24 h at 37°C from a starting OD_600_ of 0.05 in 100 µl of M63B1_Gluc_ containing 25 µg ml^−1^ of a specific amino acid if required. These bacterial cultures were moved by pipetting to new microtiter plates in order to have only the planktonic population and were then harvested from 12 wells in triplicate by centrifugation and resuspended in 100 µl of M63B1_Gluc_ containing either ticarcillin (100 µg ml^−1^) or ofloxacin (5 µg ml^−1^). Antibiotic treatment was performed for 24 h at 37°C, following which the bacteria were centrifuged and washed once with M63B1 medium to remove any traces of antibiotics prior to performing dilutions to determine CFU.

### Transposon mutagenesis

*Mariner* transposon mutagenesis was performed as previously described [61]. Briefly, plasmid pSC189 was conjugated from S17-1 λPir (pSC189) into recipient strain TG1*gfp*. The resulting transconjugants were selected on LB agar plates containing the required antibiotics (Km and Cm) and approximately 10,000 transposon mutants were transferred to 96-well microtiter plates.

### Screening of the transposon library for antibiotic tolerance in biofilms

Our transposon library was used to identify mutants with increased biofilm tolerance to the antibiotics ticarcillin (100 µg ml^−1^ - 100 times the MIC) and ofloxacin (3.125 µg ml^−1^ - 50 times the MIC). Microtiter plates containing 100 µl/well of fresh LB with the required antibiotics (Km and Cm) were inoculated directly from each plate of the frozen library (in 15% glycerol) using a 96-pin replicator and incubated statically at 37°C overnight. Overnight cultures were then transferred with a 96-pin replicator to PVC microtiter plates containing 100 µl of M63B1_Gluc_ to initiate growth of static biofilms for 24 h as described above. Biofilm inoculation via a 96-pin replicator reproduced biofilms and antibiotic susceptibility profiles similar to those normalized by optical density (data not shown). Each overnight plate was duplicated for biofilm assays in order to produce a set of plates for separately assessing the antibiotic susceptibilities to ticarcillin and ofloxacin. Following the 24-h biofilm formation period, each set of transposon mutant plates containing preformed biofilms was treated with either ticarcillin or ofloxacin in fresh M63B1_Gluc_ medium for 24 h as mentioned earlier. Antibiotic susceptibility of each mutant towards the two antibiotics was determined by colorimetric differences from the wild-type parent using the XTT-reduction assay described below. Transposon mutants displaying a 2-fold increase in tolerance were first chosen for further characterization, but due to the relatively low number of such mutants, we subsequently decreased our selection criteria to 1.5- and 1.25-fold tolerance increase to ticarcillin and ofloxacin, respectively. Using these arbitrary cut-off values likely led to the selection of false positive, however the antibiotic hypertolerance of all selected mutants based on these values were carefully validated by first transducing the mutations back into WT strain TG1 by P1*vir* prior to mapping their localization within the genome by arbitrary PCR.

### Arbitrary PCR

Transposon insertion sites were determined as previously described [62]. Briefly, this method involves a first round of PCR using a primer specific for the right end of the transposon (IR2) and an arbitrary primer (ARB1 or ARB6). A second PCR was then performed on the product from the first PCR using a primer specific to the rightmost end of the transposon (IR2-60-5) and a primer identical to the 5′ end of the arbitrary primer (ARB2). Arbitrary PCR primer sequences are listed in Table S2.

### Test of amino acid auxotrophy

One ml of an overnight LB culture of each putative auxotroph was washed twice in M63B1_Gluc_ to remove all excess of amino acids and the resuspended pellets were used to inoculate either a minimal medium liquid culture (M63B1_Gluc_) or an agar plate of M63B1_Gluc_. Growth was monitored from an overnight incubation at 37°C. Absence of growth suggested amino acid auxotrophy, which was confirmed by the addition of the corresponding amino acid at 25 µg ml^−1^ for growth restoration in minimal medium (M63B1_Gluc_) using both liquid and agar plate cultures.

### Construction of deletion mutant strains

The different *E. coli* mutant strains used in this study originated either from the Keio Collection [63], transferred to the appropriate genetic background by P1*vir* phage transduction, or were directly constructed using the λ-red linear DNA gene inactivation method [64], [65]. When required, kanamycin resistance markers flanked by two FRT sites were removed using Flp recombinase [66]. Primers used in this study are listed in Table S2. All mutations were confirmed by PCR and/or sequence analysis.

### Transcriptional and translational analysis of the SOS response

#### Transcriptional analysis in biofilms

ß-galactosidase enzymatic activity was assessed to determine the induction of the SOS response under various conditions using the reporter strain p*sulA*::*lacZ* F'tet. Biofilms were formed on the sides of microtiter wells as described above. Briefly, the reporter strain p*sulA*::*lacZ* F'tet was initially grown in LB medium with required antibiotic(s), diluted into M63B1_Gluc_ to an OD_600_ of 0.05 and used as inoculum. Each microtiter well was then inoculated with 100 µl of the OD_600_ 0.05 inoculum, with a minimum of three wells and incubated at 37°C for 24, 48, 72 or 96 hours with renewal of growth medium every 24 h. To determine the expression of *sulA* in aging biofilms, planktonic bacteria were removed from each well and the ß-galactosidase activity was directly measured on the biofilm bacteria as previously described [67].

#### Transcriptional analysis of planktonic bacteria

As described above, planktonic bacteria sharing the same environment (microtiter well) as biofilm cells were used to determine the expression of the SOS response. Growth conditions were the same as those described above for biofilm growth, but instead of using the attached bacterial cells (biofilm), the free floating cells (planktonic – 24 h) from three independent pools of 12 wells were collected for the analysis. Bacteria from the planktonic population were then harvested by centrifugation, washed, and resuspended into 100 µl of M63B1 in which the ß-galactosidase activity was directly measured as previously described [67].

#### Translational analysis

Biofilms of a reporter strain MG1655KmRExTet*lacZ_*F'tet were grown M63B1_Gluc_ for 24 h as described above. This reporter strain contains a *lacZ* gene under the control of an inducible promoter (P_LtetO-1_) by the addition of anhydrotetracycline (aTc) [68]. To determine whether *lacZ* was translated in conditions of starvation, 24-h biofilms were washed and exposed to 100 µl per well of M63B1_Gluc_ (control) or M63B1 (glucose starvation) with increasing concentration of aTc (0, 5, 50 ng ml^−1^) during a period of 1 h after which biofilms were washed and resuspended in M63B1. Crude protein extracts were prepared and equivalent amounts of proteins were first loaded on a SDS-PAGE gel, proteins were transferred to a polyvinylidene difluoride membrane, and ß-galactosidase immunodetection was performed using a 1∶10,000 dilution of mouse antiserum raised against ß-galactosidase. A planktonic culture of the same reporter strain was used as a control to verify the efficiency of the induction system in non-starving conditions.

### Statistical analysis

Analyses were performed using Prism 5.0 for Mac OS X (GraphPad Software, Inc.). Each experiment was performed at least three times.

## Supporting Information

Figure S1 Strong biofilm formation displayed by *E. coli* K-12 strain TG1. Bacterial cells of strain TG1 previously grown in LB were diluted to an OD_600_ of 0.05 in minimal medium M63B1_Gluc_, inoculated into PVC microtiter plates (100 µl/well) and incubated at 37°C for 24 h. (A) Images of PVC wells representing 24 h TG1 biofilms revealed by dissolution of the attached biomass previously stained by crystal violet (CV) as described in Materials and Methods. (B) Quantification of 24 h biofilms measuring the quantity of dissolved CV previously bound by biofilms. The high propensity of strain TG1 to form biofilm in this *in vitro* setting was demonstrated by spectrophotometric analysis at 570 nm.
(TIF)

Figure S2 Leucine starvation leads to high antibiotic tolerance in biofilms of *E. coli* TG1. The impact of leucine deprivation on biofilm-associated antibiotic tolerance was evaluated using a leucine auxotroph (Δ*leuC* – white bars) and its wild-type (WT – dark bars) prototroph TG1. Biofilms were grown for 24 h in M63B1_Gluc_ for the WT and with the addition of 25 µg ml^−1^ of leucine for the auxotroph. Biofilms were treated for 24 h with (A) ticarcillin (100 µg ml^−1^; 100× MIC) or (B) ofloxacin (5 µg ml^−1^, 80× MIC) in M63B1_Gluc_ containing different leucine concentrations. Survivors were quantified by viable cell counts. Percent survival represents the tolerant population after 24 h of treatment compared to untreated biofilm prior to addition of antibiotics. All compared biofilms had similar numbers of CFUs prior to antibiotic treatment (data not shown). Data represented are means ± SEM of at least three replicates. Asterisks indicate values significantly different from biofilms in the absence of either leucine or glucose by the two-tailed unpaired t-test: * *P*≤0.05, ** *P*≤0.01, *** *P*≤0.0001, and n.s. (not significant). The genotype of the leucine auxotroph mutant strain used is TG1Δ*leuC*::GB.
(TIF)

Figure S3 The stringent response is partially implicated in ofloxacin biofilm-specific hypertolerance. The impact of stringent response loss-of-function mutation Δ*relA* on the ofloxacin tolerance of biofilms starved for glucose (A) and leucine (B) was evaluated. Briefly, biofilms were grown for 24 h in M63B1_Gluc_ for all prototrophic strains and with the addition of 25 µg ml^−1^ of leucine for the corresponding auxotrophic strains. All biofilms were treated for 24 h in M63B1_Gluc_ containing ofloxacin (5 µg ml^−1^). For the glucose starvation environment, M63B1 without glucose was used instead of M63B1_Gluc_ (Panel A - dark bars). Partial ofloxacin sensitivity was restored by Δ*relA* in biofilms starved for leucine but not glucose when compared to non-starved biofilms. Viable cells of the treated biofilm population were quantified by viable cell counts. Percent survival represents the tolerant population after 24 h of treatment compared to untreated biofilm prior to addition of antibiotics. All compared biofilms had similar numbers of CFUs prior to antibiotic treatment (data not shown). Data represented are means ± SEM of at least three replicates. The genotypes of all the strains used here are described in Table S1 or as follows: WT (TG1), Δ*relA* (TG1Δ*relA*::KmFRT), Δ*leuC* (TG1Δ*leuC*::ΔFRT), Δ*leuC*Δ*relA* (TG1Δ*leuC*::ΔFRTΔ*relA*::KmFRT).
(TIF)

Figure S4 The impact of the SOS response on the ofloxacin tolerance of planktonic bacteria as compared to biofilm. Static cultures were grown for 24 h in M63B1_Gluc_ in microtiter wells. Planktonic bacteria from the 24-h static culture were removed from each well and therefore separated from the attached-biofilm cells. Planktonic bacteria were then collected, spun and washed. Both biofilms and planktonic bacteria were treated in medium containing ofloxacin (5 µg ml^−1^, 80× MIC) with glucose (white bars; M63B1_Gluc_) or not (black bars; M63B1) for 24 h. Survivor cells were quantified by viable cell counts. Percent survival represents the tolerant population after 24 h of treatment compared to the total number of CFU prior to addition of antibiotics. Equivalent CFU were present in all compared planktonic populations and in all biofilm population before antibiotic treatment. Data represented are means ± SEM of at least three replicates. Asterisk indicates values significantly different by the two-tailed unpaired t test: * *P*≤0.05 and n.s. (not significant). The genotypes the strains used are are described in Table S1 or as follows: WT (TG1) and Δ*recA* (TG1Δ*recA*::KmFRT).
(TIF)

Figure S5 The impact of starvation on translation efficiency. The influence of starvation on the production of β-galactosidase in 24-h biofilms of an *E. coli* strain containing a promoter P_LtetO-1_ inducible by aTc. Twenty-four-h biofilms were exposed to various concentration of aTc (0, 5, 50 ng ml^−1^) for one hour in M63B1_Gluc_ (control) or M63B1 (glucose starvation). Following the one-hour exposure, crude protein extracts were prepared and analysed by immunodetection for β-galactosidase protein detection. An exponential planktonic culture was used as a positive control of the system.
(TIF)

Figure S6 Toxin-antitoxin modules do not contribute to biofilm-associated increased ofloxacin tolerance upon leucine starvation. (A) The impact of the four SOS-TA modules on ofloxacin tolerance in biofilms upon starvation to leucine (Δ*leuC*) compared to no starvation (WT). SOS-Dep TA (+) strains in WT prototrophic (WT; MG1655Δ*lac*F'tet) and leucine auxotroph (Δ*leuC*; MG1655F'tetΔ*lac*Δ*leuC*::KmFRT) backgrounds were used. MG1655Δ*lac*F'tet (WT; SOS-Dep TA (+)) and its leucine auxotroph MG1655F'tetΔ*lac*Δ*leuC*::KmFRT (Δ*leuC*; TA (+)) were compared to their respective negative SOS-Dep TA strains (TA (−)). (B) The impact of non-SOS-TA modules as well as Lon protease on biofilm-associated ofloxacin tolerance upon starvation to leucine (5 µg ml^−1^, 80× MIC) for 24 h and survivor cells were quantified by viable cell counts. Non-starved biofilms of WT TG1 were compared to biofilms starved for leucine using auxotrophic strains to leucine (Δ*leuC*::ΔFRT) deficient in various TA loci or Lon. Percent survival represents viable cells after 24 h of treatment compared to untreated biofilm prior to addition of antibiotics. Data represented are means ± SEM of at least three replicates. All strains used for panel B were made in a TG1 genetic background and are described in Table S1 or as follows: Δ*leuC* (Δ*leuC*::ΔFRT), Δ*relE* (Δ*leuC*:: ΔFRTΔ*relE*::KmFRT), Δ*mazF* (Δ*leuC*::ΔFRTΔ*mazF*::KmFRT), Δ*hipA* (Δ*leuC*::ΔFRTΔ*hipA*::KmFRT), Δ*chpB* (Δ*leuC*::ΔFRTΔ*chpB*::KmFRT), Δ*hicA* (Δ*leuC*::ΔFRTΔ*hicA*::KmFRT), Δ*yoeB* (Δ*leuC*::ΔFRTΔ*yoeB*::KmFRT), and Δ*ccdB* (Δ*leuC*::GBΔ*ccdB*::Spec).
(TIF)

Table S1 Bacterial strains and plasmids used in this study.
(DOCX)

Table S2 Oligonucleotide primers used in this study.
(DOCX)
