# Supplementary figures and images for: Starvation, Together with the SOS Response, Mediates High Biofilm-Specific Tolerance to the Fluoroquinolone Ofloxacin
Source: PLoS Genet. 2013 Jan 3;9(1):e1003144. doi: 10.1371/journal.pgen.1003144 (PMC3536669; doi:10.1371/journal.pgen.1003144)

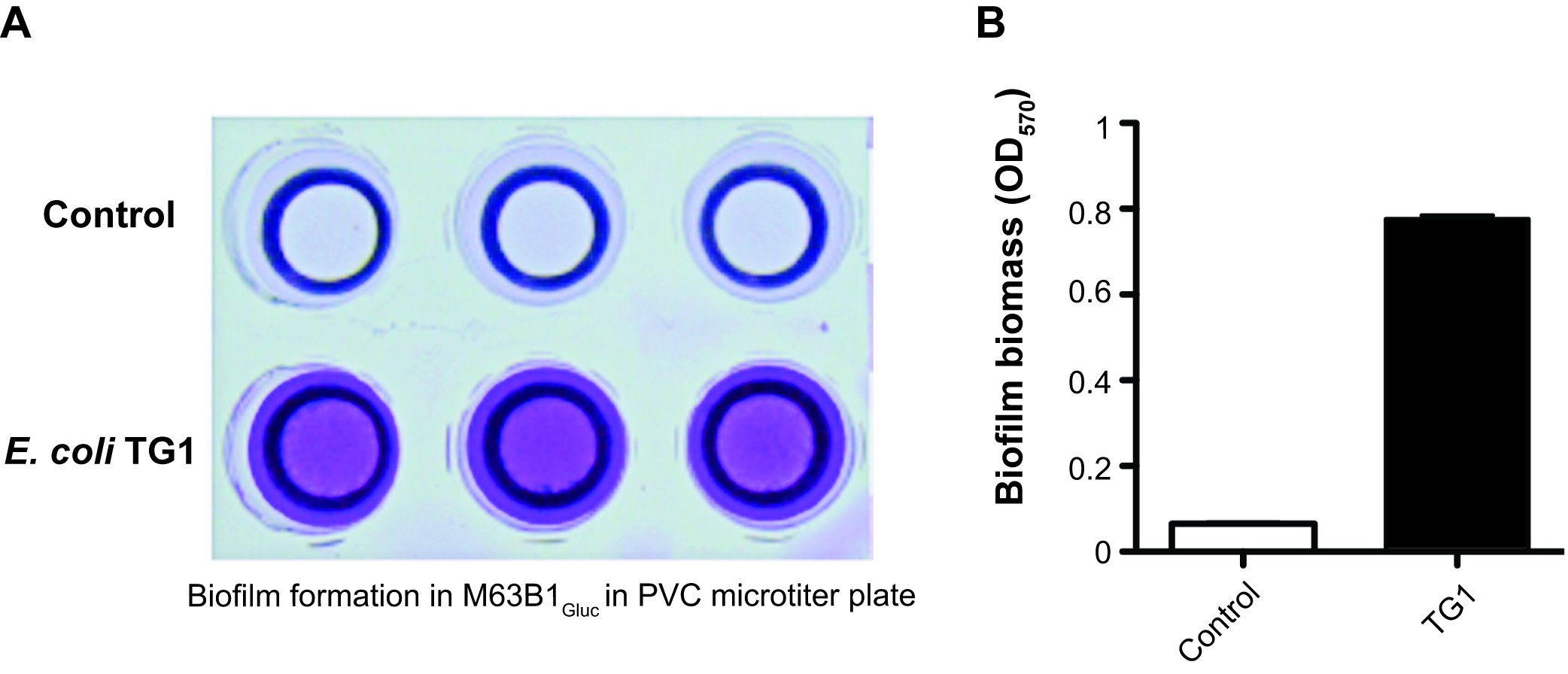

Supplement: Figure S1 — Strong biofilm formation displayed by E. coli K-12 strain TG1. Bacterial cells of strain TG1 previously grown in LB were diluted to an OD600 of 0.05 in minimal medium M63B1Gluc, inoculated into PVC microtiter plates (100 µl/well) and incubated at 37°C for 24 h. (A) Images of PVC wells representing 24 h TG1 biofilms revealed by dissolution of the attached biomass previously stained by crystal violet (CV) as described in Materials and Methods. (B) Quantification of 24 h biofilms measuring the quantity of dissolved CV previously bound by biofilms. The high propensity of strain TG1 to form biofilm in this in vitro setting was demonstrated by spectrophotometric analysis at 570 nm. (TIF) [file pgen.1003144.s001.tif]

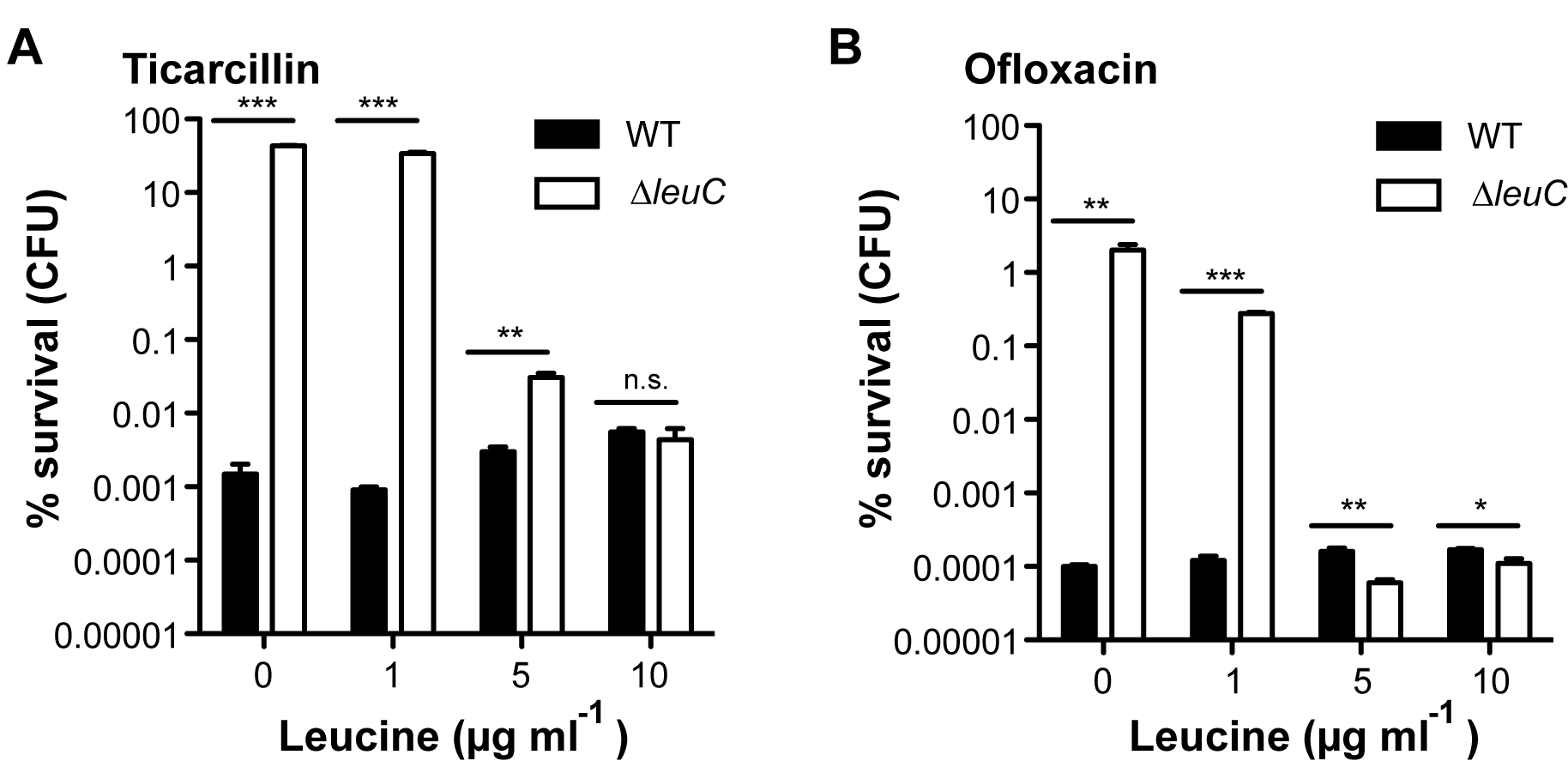

Supplement: Figure S2 — Leucine starvation leads to high antibiotic tolerance in biofilms of E. coli TG1. The impact of leucine deprivation on biofilm-associated antibiotic tolerance was evaluated using a leucine auxotroph (ΔleuC – white bars) and its wild-type (WT – dark bars) prototroph TG1. Biofilms were grown for 24 h in M63B1Gluc for the WT and with the addition of 25 µg ml−1 of leucine for the auxotroph. Biofilms were treated for 24 h with (A) ticarcillin (100 µg ml−1; 100× MIC) or (B) ofloxacin (5 µg ml−1, 80× MIC) in M63B1Gluc containing different leucine concentrations. Survivors were quantified by viable cell counts. Percent survival represents the tolerant population after 24 h of treatment compared to untreated biofilm prior to addition of antibiotics. All compared biofilms had similar numbers of CFUs prior to antibiotic treatment (data not shown). Data represented are means ± SEM of at least three replicates. Asterisks indicate values significantly different from biofilms in the absence of either leucine or glucose by the two-tailed unpaired t-test: * P≤0.05, ** P≤0.01, *** P≤0.0001, and n.s. (not significant). The genotype of the leucine auxotroph mutant strain used is TG1ΔleuC::GB. (TIF) [file pgen.1003144.s002.tif]

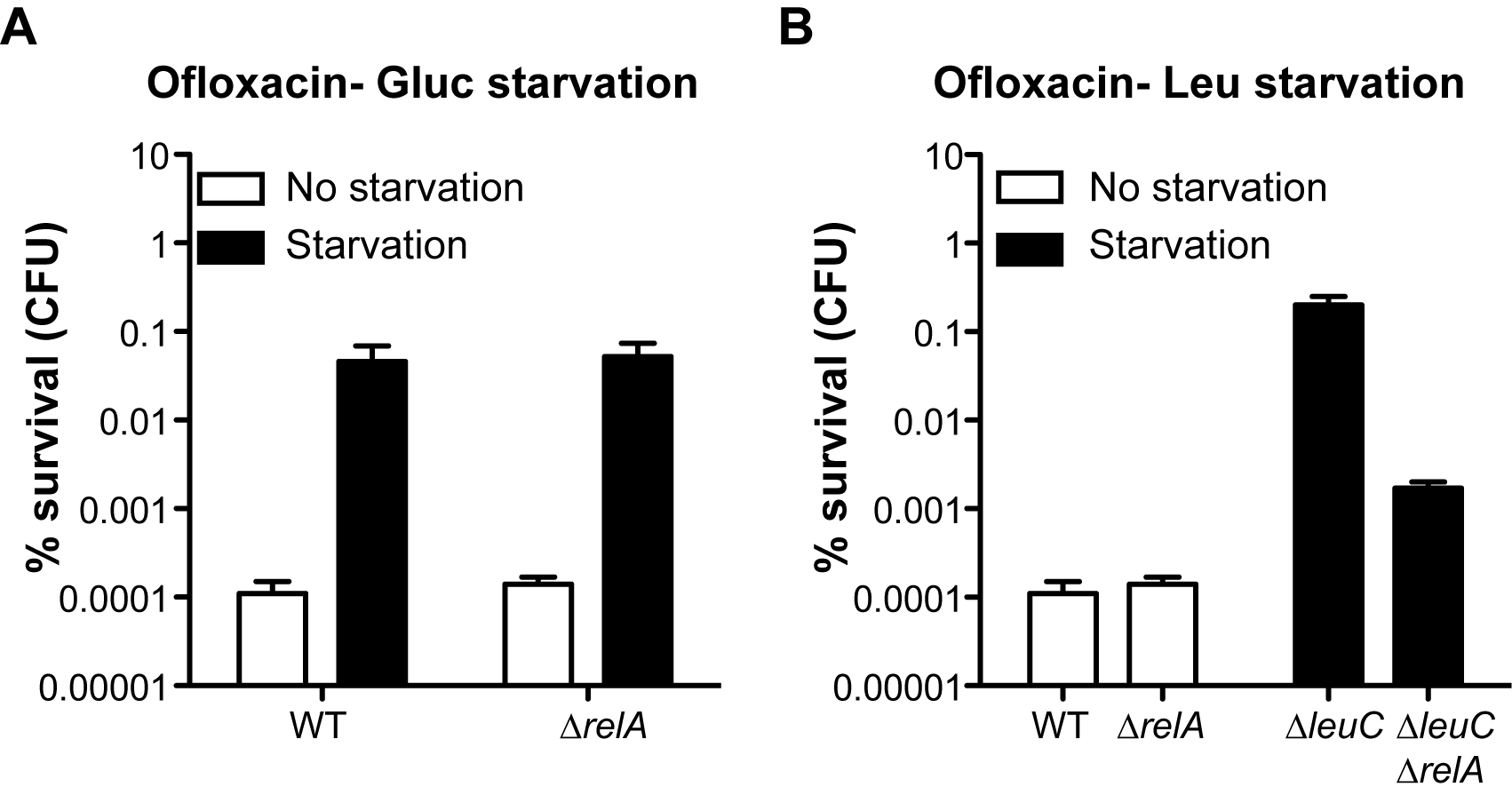

Supplement: Figure S3 — The stringent response is partially implicated in ofloxacin biofilm-specific hypertolerance. The impact of stringent response loss-of-function mutation ΔrelA on the ofloxacin tolerance of biofilms starved for glucose (A) and leucine (B) was evaluated. Briefly, biofilms were grown for 24 h in M63B1Gluc for all prototrophic strains and with the addition of 25 µg ml−1 of leucine for the corresponding auxotrophic strains. All biofilms were treated for 24 h in M63B1Gluc containing ofloxacin (5 µg ml−1). For the glucose starvation environment, M63B1 without glucose was used instead of M63B1Gluc (Panel A - dark bars). Partial ofloxacin sensitivity was restored by ΔrelA in biofilms starved for leucine but not glucose when compared to non-starved biofilms. Viable cells of the treated biofilm population were quantified by viable cell counts. Percent survival represents the tolerant population after 24 h of treatment compared to untreated biofilm prior to addition of antibiotics. All compared biofilms had similar numbers of CFUs prior to antibiotic treatment (data not shown). Data represented are means ± SEM of at least three replicates. The genotypes of all the strains used here are described in Table S1 or as follows: WT (TG1), ΔrelA (TG1ΔrelA::KmFRT), ΔleuC (TG1ΔleuC::ΔFRT), ΔleuCΔrelA (TG1ΔleuC::ΔFRTΔrelA::KmFRT). (TIF) [file pgen.1003144.s003.tif]

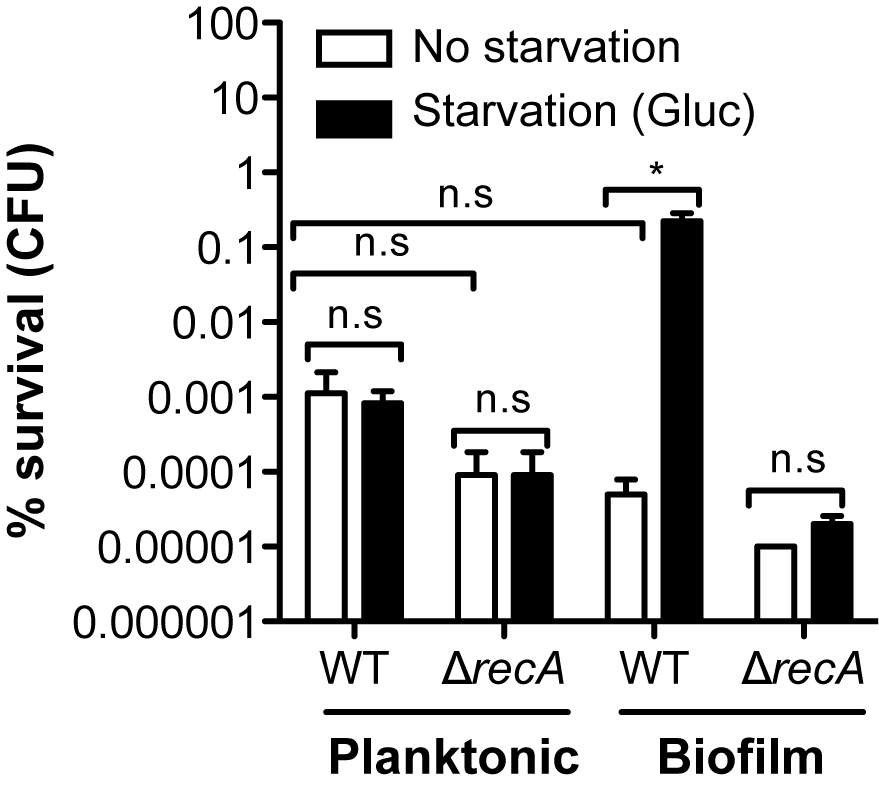

Supplement: Figure S4 — The impact of the SOS response on the ofloxacin tolerance of planktonic bacteria as compared to biofilm. Static cultures were grown for 24 h in M63B1Gluc in microtiter wells. Planktonic bacteria from the 24-h static culture were removed from each well and therefore separated from the attached-biofilm cells. Planktonic bacteria were then collected, spun and washed. Both biofilms and planktonic bacteria were treated in medium containing ofloxacin (5 µg ml−1, 80× MIC) with glucose (white bars; M63B1Gluc) or not (black bars; M63B1) for 24 h. Survivor cells were quantified by viable cell counts. Percent survival represents the tolerant population after 24 h of treatment compared to the total number of CFU prior to addition of antibiotics. Equivalent CFU were present in all compared planktonic populations and in all biofilm population before antibiotic treatment. Data represented are means ± SEM of at least three replicates. Asterisk indicates values significantly different by the two-tailed unpaired t test: * P≤0.05 and n.s. (not significant). The genotypes the strains used are are described in Table S1 or as follows: WT (TG1) and ΔrecA (TG1ΔrecA::KmFRT). (TIF) [file pgen.1003144.s004.tif]

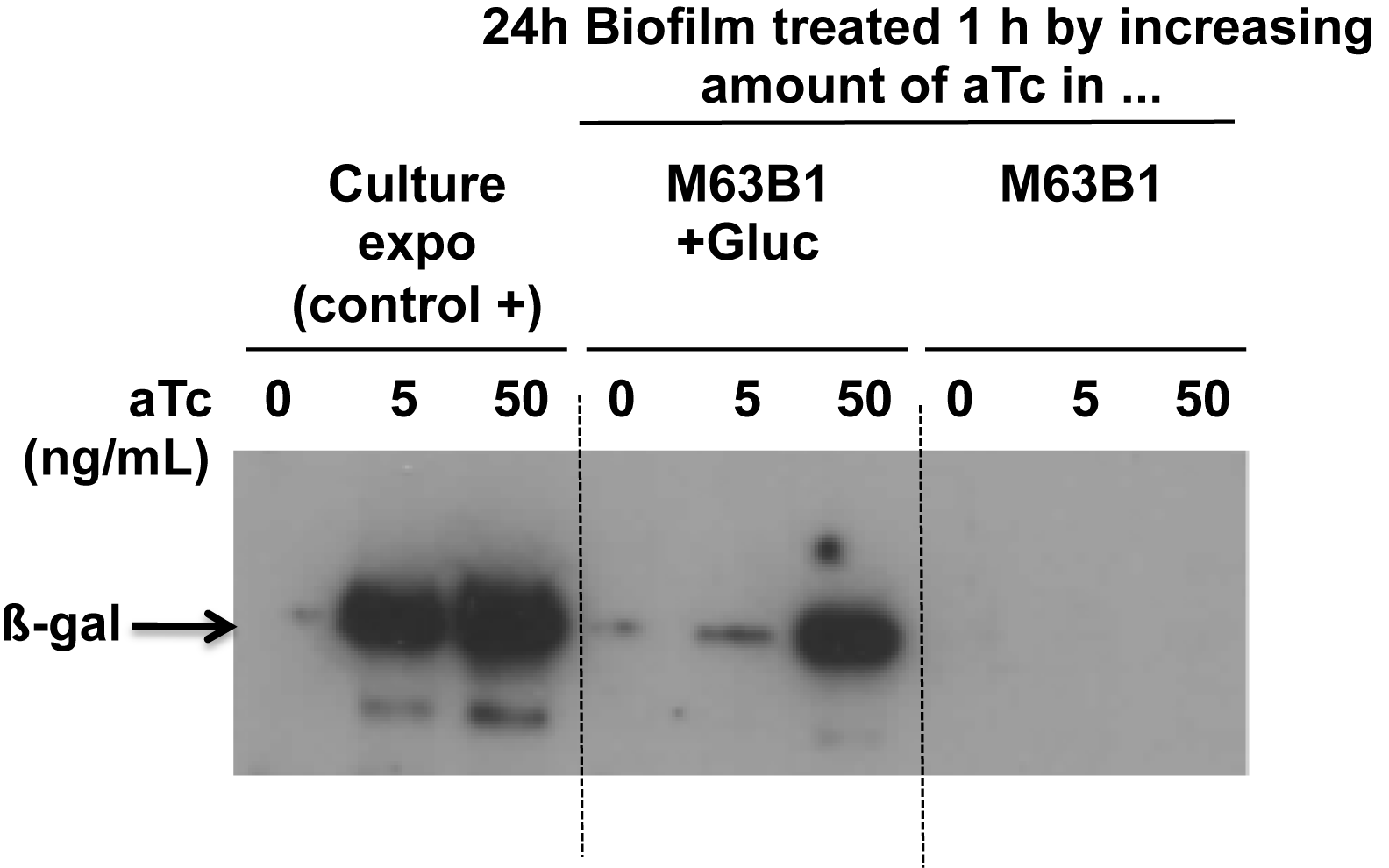

Supplement: Figure S5 — The impact of starvation on translation efficiency. The influence of starvation on the production of β-galactosidase in 24-h biofilms of an E. coli strain containing a promoter PLtetO-1 inducible by aTc. Twenty-four-h biofilms were exposed to various concentration of aTc (0, 5, 50 ng ml−1) for one hour in M63B1Gluc (control) or M63B1 (glucose starvation). Following the one-hour exposure, crude protein extracts were prepared and analysed by immunodetection for β-galactosidase protein detection. An exponential planktonic culture was used as a positive control of the system. (TIF) [file pgen.1003144.s005.tif]

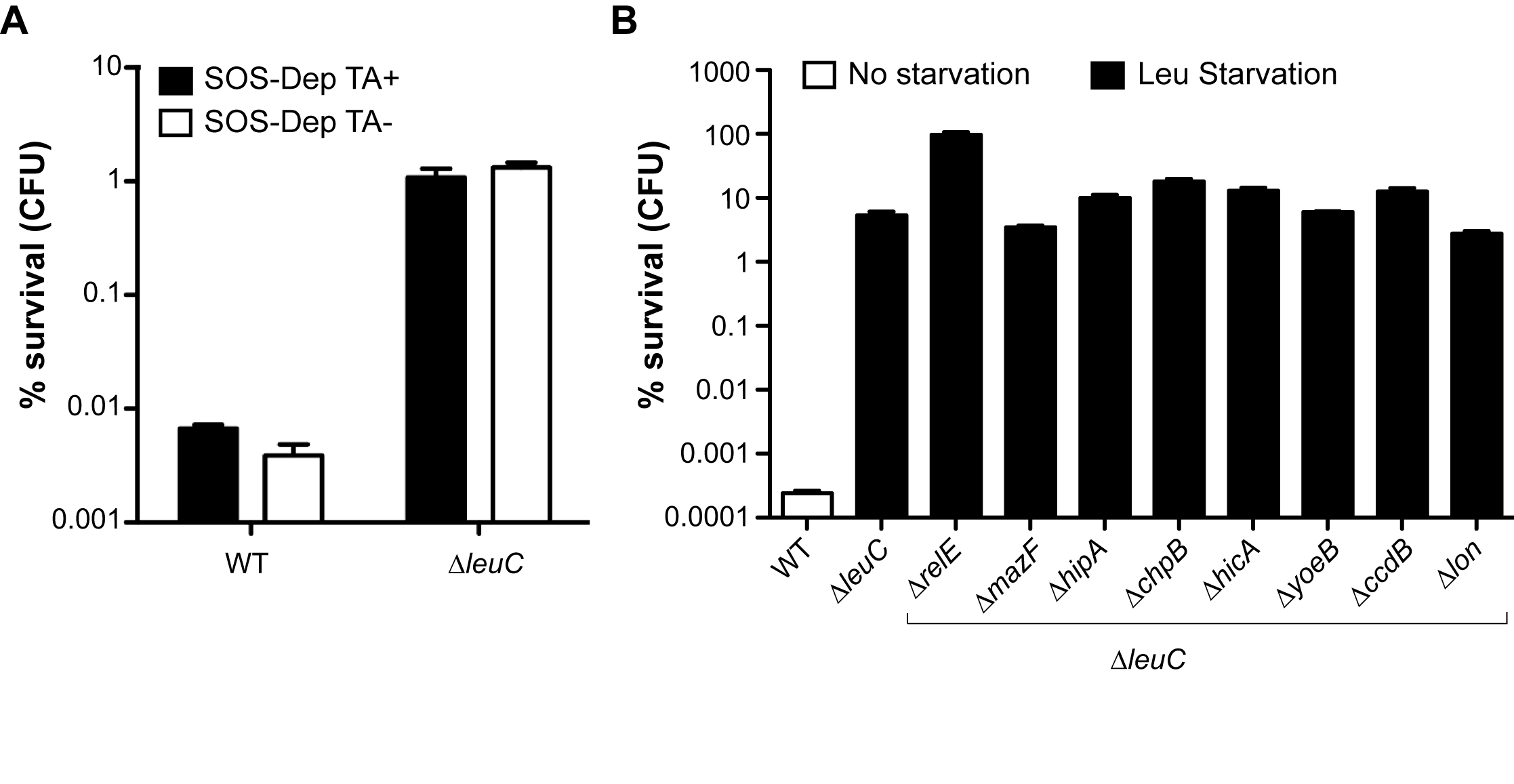

Supplement: Figure S6 — Toxin-antitoxin modules do not contribute to biofilm-associated increased ofloxacin tolerance upon leucine starvation. (A) The impact of the four SOS-TA modules on ofloxacin tolerance in biofilms upon starvation to leucine (ΔleuC) compared to no starvation (WT). SOS-Dep TA (+) strains in WT prototrophic (WT; MG1655ΔlacF'tet) and leucine auxotroph (ΔleuC; MG1655F'tetΔlacΔleuC::KmFRT) backgrounds were used. MG1655ΔlacF'tet (WT; SOS-Dep TA (+)) and its leucine auxotroph MG1655F'tetΔlacΔleuC::KmFRT (ΔleuC; TA (+)) were compared to their respective negative SOS-Dep TA strains (TA (−)). (B) The impact of non-SOS-TA modules as well as Lon protease on biofilm-associated ofloxacin tolerance upon starvation to leucine (5 µg ml−1, 80× MIC) for 24 h and survivor cells were quantified by viable cell counts. Non-starved biofilms of WT TG1 were compared to biofilms starved for leucine using auxotrophic strains to leucine (ΔleuC::ΔFRT) deficient in various TA loci or Lon. Percent survival represents viable cells after 24 h of treatment compared to untreated biofilm prior to addition of antibiotics. Data represented are means ± SEM of at least three replicates. All strains used for panel B were made in a TG1 genetic background and are described in Table S1 or as follows: ΔleuC (ΔleuC::ΔFRT), ΔrelE (ΔleuC:: ΔFRTΔrelE::KmFRT), ΔmazF (ΔleuC::ΔFRTΔmazF::KmFRT), ΔhipA (ΔleuC::ΔFRTΔhipA::KmFRT), ΔchpB (ΔleuC::ΔFRTΔchpB::KmFRT), ΔhicA (ΔleuC::ΔFRTΔhicA::KmFRT), ΔyoeB (ΔleuC::ΔFRTΔyoeB::KmFRT), and ΔccdB (ΔleuC::GBΔccdB::Spec). (TIF) [file pgen.1003144.s006.tif]
