# Supplementary material for: Starvation, Together with the SOS Response, Mediates High Biofilm-Specific Tolerance to the Fluoroquinolone Ofloxacin
Source: PLoS Genet. 2013 Jan 3;9(1):e1003144. doi: 10.1371/journal.pgen.1003144 (PMC3536669; doi:10.1371/journal.pgen.1003144)
Supplement: Table S1 — Bacterial strains and plasmids used in this study. (DOCX) [file pgen.1003144.s007.docx]

**Table S1. Bacterial strains and plasmids used in this study.**

| **Strain** | Relevant characteristics | | Reference/source |  |
| --- | --- | --- | --- | --- |
| TG1 | Wild-type *E. coli* K12; F'(*traD*36 *proAB* + *lacI^q^* *lacZ*ΔM15 *supE hsd*Δ5 *thi* Δ(*lac-proAB*)) | | Laboratory collection |  |
| MG1655 | Wild-type *E. coli* K12 | | Laboratory collection |  |
| S17-1λPir | RP4-2Tc::Mu Km::Tn7 λpir; Pir-dependent replication | | [[1](#_ENREF_1)] |  |
| TG1 derivatives |  | |  |  |
| TG1*gfp* | Insertion, at the λatt site, of *gfp* gene variant Mut3 under control of the constitutive λ*p*_R_ promoter; Cm*^R^* | | Sandra Da Re |  |
| ∆*leuC* | TG1∆*leuC*::GB; leucine auxotroph; Km^R^ | | This study |  |
| ∆*leuC*::KmFRT | TG1∆*leuC*::KmFRT; mutation obtained from JW0071^a^; leucine auxotroph; Km^R^ | | This study |  |
| ∆*leuC*::∆FRT | TG1∆*leuC*::∆FRT; deletion of the Km^R^ by the *flp* recombinase; leucine auxotroph | | This study |  |
| 36B6*leuC* | Insertion of pSC189 into *leuC*; leucine auxotroph; Km^R^ | | This study |  |
| 39D8*leuC* | Insertion of pSC189 into *leuC*; leucine auxotroph; Km^R^ | | This study |  |
| 91G7*leuC* | Insertion of pSC189 into *leuC*; Leucine auxotroph; Km^R^ | | This study |  |
| 104H7*leuC* | Insertion of pSC189 into *leuC*; leucine auxotroph; Km^R^ | | This study |  |
| *leu-82* | TG1*leu-82*::Tn*10*^b^; Tet^R^ | | This study |  |
| ∆*hisG* | TG1∆*hisG*::KmFRT; mutation obtained from JW2001^a^; histidine auxotroph; Km^R^ | | This study |  |
| ∆*pheA* | TG1∆*pheA*::KmFRT; mutation obtained from JW2580^a^; phenylalanine auxotroph; Km^R^ | | This study |  |
| ∆*argH* | TG1∆*argH*::KmFRT; mutation obtained from JW3932^a^; arginine auxotroph; Km^R^ | | This study |  |
| ∆*ilvA* | TG1∆*ilvA*::KmFRT; mutation obtained from JW3745^a^; isoleucine auxotroph; Km^R^ | | This study |  |
| ∆*proC* | TG1∆*proC*::KmFRT; mutation obtained from JW0377^a^; proline auxotroph; Km^R^ | | This study |  |
| ∆*tyrA* | TG1∆*tyrA*::KmFRT; mutation obtained from JW2581^a^; tyrosine auxotroph; Km^R^ | | This study |  |
| ∆*trpA* | TG1∆*trpA*::KmFRT; mutation obtained from JW1252^a^; tryptophan auxotroph; Km^R^ | | This study |  |
| ∆*cysD* | TG1∆*cysD*::KmFRT; mutation obtained from JW2722^a^; cysteine auxotroph; Km^R^ | | This study |  |
| ∆*lysA* | TG1∆*lysA*::KmFRT; mutation obtained from JW2806^a^; lysine auxotroph; Km^R^ | | This study |  |
| ∆*metA* | TG1∆*metA*::KmFRT; mutation obtained from JW3973^a^; methionine auxotroph; Km^R^ | | This study |  |
| ∆*recA* | TG1∆*recA*::KmFRT; mutation obtained from JW2669^a^; Km^R^ | | This study |  |
| *lexAind3* | TG1*lexAind3malF*::Tn*10*^c^; Tet^R^, uncleavable LexA, tested for UV^S^ | | This study |  |
| ∆*relA* | TG1∆*relA*::KmFRT; mutation obtained from JW2755^a^; Km^R^ | | This study |  |
| ∆*leuC*∆*recA* | TG1∆*leuC*::∆FRT∆*recA*::KmFRT; leucine auxotroph; Km^R^ | | This study |  |
| ∆*leuC lexAind3* | TG1∆*leuC*::∆FRT*lexAind3malF*::Tn*10*; uncleavable LexA, tested for UV^S^, leucine auxotroph; Tet^R^ Km^R^ | | This study |  |
| ∆*leuC*∆*relA* | TG1∆*leuC*::∆FRT∆*relA*::KmFRT; mutation ∆*relA* obtained from JW2755^a^; leucine auxotroph; Km^R^ | | This study |  |
| ∆*lysA*∆*recA* | TG1∆*lysA*::∆FRT∆*recA*::KmFRT; lysine auxotroph; Km^R^ | | This study |  |
| ∆*trpA*∆*recA* | TG1∆*trpA*::∆FRT∆*recA*::KmFRT; tryptophan auxotroph; Km^R^ | | This study |  |
| ∆*leuC*∆*ccdB* | TG1∆*leuC*::KmGB∆*ccdB*::Spec; leucine auxotroph; Km^R^, Spec^R^ | | This study |  |
| ∆*leuC*∆*hipA* | TG1∆*leuC*::∆FRT∆*hipA*::KmFRT; mutation ∆*hipA* obtained from JW1500^a^; leucine auxotroph; Km^R^ | | This study |  |
| ∆*leuC*∆*hicA* | TG1∆*leuC*::∆FRT∆*hicA*::KmFRT; mutation ∆*hicA* obtained from JW5230^a^; leucine auxotroph; Km^R^ | This study | | |
| ∆*leuC*∆*yoeB* | TG1∆*leuC*::∆FRT∆*yoeB*::KmFRT; mutation ∆*yoeB* obtained from JW5331^a^; leucine auxotroph; Km^R^ | This study | | |
| ∆*leuC*∆*chpB* | TG1∆*leuC*::∆FRT∆*chpB*::KmFRT; mutation ∆*chpB* obtained from JW4184^a^; leucine auxotroph; Km^R^ | This study | | |
| ∆*leuC*∆*mazF* | TG1∆*leuC*::∆FRT∆*mazF*::KmFRT; mutation ∆*mazF* obtained from JW2753^a^; leucine auxotroph; Km^R^ | This study | | |
| ∆*leuC*∆*relE* | TG1∆*leuC*::∆FRT∆*relE*::KmFRT; mutation ∆*relE* obtained from JW1555^a^; leucine auxotroph; Km^R^ | This study | | |
| ∆*leuC*∆*lon* | TG1∆*leuC*::∆FRT∆*lon*::KmFRT; mutation ∆*lon* obtained from JW0429^a^; leucine auxotroph; Km^R^ | This study | | |
| ∆*serA* | TG1∆*serA*::KmFRT; mutation obtained from JW2880^a^; serine auxotroph; Km^R^ | | This study |  |
| ∆*thrA* | TG1∆*thrA*::KmFRT; mutation obtained from JW0001^a^; threonine auxotroph; Km^R^ | | This study |  |
| ∆*glyA* | TG1∆*glyA*::KmFRT; mutation obtained from JW2535^a^; glycine auxotroph; Km^R^ | | This study |  |
| ∆*glnA* | TG1∆*glnA*::KmFRT; mutation obtained from JW3841^a^; glutamine auxotroph; Km^R^ | | This study |  |
| ∆*gltA* | TG1∆*gltA*::KmFRT; mutation obtained from JW0710^a^; glutamic acid auxotroph; Km^R^ | | This study |  |
| TG1*gfp* derivatives |  | |  |  |
| 36B6-GFP*leuC* | Insertion of pSC189 into *leuC*; leucine auxotroph; Km^R^ and Cm^R^ | | This study |  |
| 39D8-GFP*leuC* | Insertion of pSC189 into *leuC*; leucine auxotroph; Km^R^ and Cm^R^ | | This study |  |
| 91G7-GFP*leuC* | Insertion of pSC189 into *leuC*; leucine auxotroph; Km^R^ and Cm^R^ | | This study |  |
| 104H7-GFP*leuC* | Insertion of pSC189 into *leuC*; leucine auxotroph; Km^R^ and Cm^R^ | | This study |  |
| 50D6-GFP*leuB* | Insertion of pSC189 into *leuB*; leucine auxotroph; Km^R^ and Cm^R^ | | This study |  |
| 44F5-GFP*aroE* | Insertion of pSC189 into *aroE*; aromatic amino acid auxotroph; Km^R^ and Cm^R^ | | This study |  |
| 70C4-GFP*argE* | Insertion of pSC189 into *argE*; arginine auxotroph; Km^R^ and Cm^R^ | | This study |  |
| 47E6-GFP*ilvC* | Insertion of pSC189 into *ilvC*; isoleucine/valine auxotrophs; Km^R^ and Cm^R^ | | This study |  |
| 79E4-GFP*proA* | Insertion of pSC189 into *proA*; proline auxotroph; Km^R^ and Cm^R^ | | This study |  |
| 94H3-GFP*proA* | Insertion of pSC189 into *proA*; proline auxotroph; Km^R^ and Cm^R^ | | This study |  |
| 84C10-GFP | Unknown insertion of pSC189; threonine auxotroph; Km^R^ and Cm^R^ | | This study |  |
| 17E11-GFP | Unknown insertion of pSC189; threonine auxotroph; Km^R^ and Cm^R^ | | This study |  |
| 57A6-GFP | Unknown insertion of pSC189; histidine auxotroph; Km^R^ and Cm^R^ | | This study |  |
| 3H4-GFP | Unknown insertion of pSC189; proline auxotroph; Km^R^ and Cm^R^ | | This study |  |
| 102A7-GFP | Unknown insertion of pSC189; tyrosine auxotroph; Km^R^ and Cm^R^ | | This study |  |
| 84A12-GFP | Unknown insertion of pSC189; cysteine auxotroph; Km^R^ and Cm^R^ | | This study |  |
| 104D4-GFP*pnp* | Insertion of pSC189 into *pnp*; Km^R^ and Cm^R^ | | This study |  |
| 19F12-GFP*rseC* | Insertion of pSC189 into *rseC*; Km^R^ and Cm^R^ | | This study |  |
| MG1655 derivatives |  | |  |  |
| *Km*PcL*fim* | MG1655*Km*PcL*fim*, strain constitutively expressing type 1 fimbriae; strong biofilm former; Km^R^ | | [[2](#_ENREF_2)] |  |
| *Km*PcL*fim* *leu-82* | MG1655*Km*PcL*fimleu-82*::Tn*10* ^b^; leucine auxotroph; Km^R^, Tet^R^ | | This study |  |
| MG1655 F’tet∆lac | MG1655 F’tet∆*lacIZ*::*cat*, Tet^R^, Cm^R^ | | This study |  |
| SOS-TA | MG1655 F’tet∆lac∆4SOS-TA (*tisAB*::∆FRT, *symER*:: ∆FRT, *dinJyafQ*::∆FRT, *yafNO*::∆FRT)^d^ | | This study |  |
| SOS-TA *leuC* | SOS-TA∆*leuC*::KmFRT, leucine auxotroph; Km^R^ | | This study |  |
| MG1655KmRExTet*lacZ* | *lacZ* with its own RBS under the control of the  kmRExTET cassette P_LtetO-1_ promoter; Km^R^ | | [[3](#_ENREF_3)] |  |
| MG1655KmRExTet*lacZ*_F'tet | conjugation of F'tet in MG1655KmRExTet*lacZ*, Km^R^, Tet^R^ | | This study |  |
| Other *E. coli* strains |  | |  |  |
| JJC610 | ∆(*lac*-*pro*) *rpsL* λatt[p*sulA*::*lacZ*], Strep^R^ | | [[4](#_ENREF_4)] |  |
| p*sulA*::*lacZ*_F'tet | Conjugation of F'tet in strain JJC610, Strep^R^, Tet^R^ | | This study |  |
| **Plasmids** |  | |  |  |
| pSC189 | Plasmid bearing the *mariner* transposon; R6K *ori*; Amp^R^ and Km^R^ | | [[5](#_ENREF_5)] |  |
| pCP20 | Plasmid bearing the *flp* recombinase gene; 30ºC replication; Cm^R^ and Amp^R^ | | [[6](#_ENREF_6)] |  |
| pAM34 | Cloning vector whose replication is dependent on the presence of 500 µM of IPTG; Spec^R^, Amp^R^ | | [[7](#_ENREF_7)] |  |
| pAM34*recA* | *recA* gene cloned in pAM34 ; Spec^R^, Amp^R^ | | [[8](#_ENREF_8)] |  |
|  |  | |  |  |

^a^ Mutation obtained from the Keio collection gene deletion library mutant [9] and transduced by P1*vir* into the desired background.

^b^ Mutation obtained from [10] and transduced by P1*vir* into the desired background.

^c^ Mutation obtained from [11] and transduced by P1*vir* into the desired background. A single TG1*malF*::Tn*10* had no effect on either ticarcillin or ofloxacin tolerance in biofilms (data not shown).

^d^ Mutation obtained from strain MG1655∆4SOS-TA (*tisAB*::ΔFRT, *symER*::ΔFRT, *dinJyafQ*::ΔFRT, *yafNO*::ΔFRT). Kindly provided by Dr L. van Melderen (unpublished). This particular strain was constructed by introducing the derepressed tetracycline-tagged F episome into MG1655∆4SOS-TA carrying all four SOS-TA deletions.

**Supporting References**

1. Simon R, Priefer U, Pühler A (1983) A broad host range mobilization system for *in vivo* genetic engineering : transposon mutagenesis in Gram negative bacteria. Biotechnology 1: 784-791.

2. Korea CG, Badouraly R, Prevost MC, Ghigo JM, Beloin C (2010) *Escherichia* *coli* K-12 possesses multiple cryptic but functional chaperone-usher fimbriae with distinct surface specificities. Environ Microbiol 12: 1957-1977.

3. Da Re S, Le Quere B, Ghigo JM, Beloin C (2007) Tight modulation of *Escherichia* *coli* bacterial biofilm formation through controlled expression of adhesion factors. Appl Environ Microbiol 73: 3391-3403.

4. Flores MJ, Sanchez N, Michel B (2005) A fork-clearing role for UvrD. Mol Microbiol 57: 1664-1675.

5. Chiang SL, Rubin EJ (2002) Construction of a mariner-based transposon for epitope-tagging and genomic targeting. Gene 296: 179-185.

6. Cherepanov PP, Wackernagel W (1995) Gene disruption in *Escherichia coli*: Tc^R^ and Km^R^ cassettes with the option of Flp-catalyzed excision of the antibiotic-resistance determinant. Gene 158: 9-14.

7. Evans DJ, Allison DG, Brown MR, Gilbert P (1991) Susceptibility of *Pseudomonas aeruginosa* and *Escherichia coli* biofilms towards ciprofloxacin: effect of specific growth rate. J Antimicrob Chemother 27: 177-184.

8. Boubakri H, de Septenville AL, Viguera E, Michel B (2010) The helicases DinG, Rep and UvrD cooperate to promote replication across transcription units *in vivo*. EMBO J 29: 145-157.

9. Baba T, Ara T, Hasegawa M, Takai Y, Okumura Y, et al. (2006) Construction of *Escherichia coli* K-12 in-frame, single-gene knockout mutants: the Keio collection. Mol Syst Biol 2: 2006 0008.

10. Sezonov G, Joseleau-Petit D, D'Ari R (2007) *Escherichia coli* physiology in Luria-Bertani broth. J Bacteriol 189: 8746-8749.

11. Bierne H, Seigneur M, Ehrlich SD, Michel B (1997) *uvrD* mutations enhance tandem repeat deletion in the *Escherichia* *coli* chromosome via SOS induction of the RecF recombination pathway. Mol Microbiol 26: 557-567.
