# Supplementary material for: Starvation, Together with the SOS Response, Mediates High Biofilm-Specific Tolerance to the Fluoroquinolone Ofloxacin
Source: PLoS Genet. 2013 Jan 3;9(1):e1003144. doi: 10.1371/journal.pgen.1003144 (PMC3536669; doi:10.1371/journal.pgen.1003144)
Supplement: Table S2 — Oligonucleotide primers used in this study. (DOCX) [file pgen.1003144.s008.docx]

**Table S2. Oligonucleotide primers used in this study.**

| Primer | Sequence 5'- 3' |
| --- | --- |
| *Arbitrary PCR* |  |
| IR2 | CTgACCgCTTCCTCgTgCTTTACgg |
| ARB1 | ggCCACgCgTCgACTAgTACNNNNNNNNNNgATAT |
| ARB6 | ggCCACgCgTCgACTAgTACNNNNNNNNNNACgCC |
| ARB2 | ggCCACgCgTCgACTAgTAC |
| IR2.60-5 | TTCTGAgcgggactctggggtacg |
| *Mutant deletion* |  |
| leuCKmGBDir-LongL5' | GCCTGTGTGTTTGATAAATTTCTCTGCCATGGTGTGCTCCTTAGAAAAACTCATCGAGCATCAA |
| leuCKmGBDir-LongL3' | GCGATATCATTGCCCGCTATGTAGCAGAAGGGGTGTAATCaaagccacgttgtgtctcaaaatc |
| leuDKmGBDir-LongL5 | ACGATAGCGGAAAGCCCGGTCATTTGACCGGGCAAGGGGATTAGAAAAACTCATCGAGCATCAA |
| leuDKmGBDir-LongL3 | CATTTCGCCGACATTCGCAACATTAAAtaaGGAGCACACCaaagccacgttgtgtctcaaaatc |
| ccdB.500-3 | gtccttcctgtgcgacggttac |
| ccdB.spec.L-5 | AAACTGCTTGGTGCCAGCCAATGAatgtcaggctccgttatacacagc |
| ccdB.500-5 | ggggcggaaaacagcgatatga |
| ccdB.spec.L-3 | gtatttattcggcgcaattgACGCtTCACCAGTCCCTGTTCTCGTCAG |
| *Mutant confirmation* |  |
| leuCD.GB.ext-3 | TCGACGTTAATGTGGACCTGCAAC |
| leuC.GB.ext-5 | AGTGCGCAATGATCACTGGCTCGATGGG |
| serA.out.5' | TTTGGTGACATGTGTCACGC |
| serA.out.3' | TCCTTCAAGCCCTCGCTTCG |
| pheA.out.5' | ACAATCAATACACCTAACGG |
| pheA.out.3' | AGCATACCAATGGTTTCTGG |
| trpA.out.5' | AGATTGAAGAATCTTACTCC |
| trpA.out.3' | ACACTCATTAAAATTAGTCG |
| cysD.out.5' | ATCGCCAGGTTTAGGTGACG |
| cysD.out.3' | AATGGTGATGCCCTGTTCGC |
| argE.out.5' | ATCGTGGCTAACTTCATGGG |
| argE.out.3' | AAAGCGGTGACGTCAAATGC |
| argH.out.5' | CTGGAGAACGGTTATCTGCC |
| argH.out.3' | CTTCCAGCTTACGAATTTGC |
| mazF.out.5' | ATCCACAGTAGCGTAAAGC |
| mazF.out.3' | ATATCATTAAAGTCAAAGCG |
| relE.out.5' | CTACTTTCTGACTTCCTTCG |
| relE.out.3' | TCTCTTACTCCTCAGGTTCG |
| yoeB.out.5' | AATGAACTGTACAAAAGAGG |
| yoeB.out.3' | ATCCAGATCGTCAGAATCGC |
| chpB.out.5' | CTTTGTATAACTTAAGGAGG |
| chpB.out.3' | CAAGCTGAGCAAAGAATACG |
| hipA.out.5' | CTTTCAGAAGATCTATAGCC |
| hipA.out.3' | CTGAACGAATAACGACTACC |
| hicA.out.5' | ACACGTTAATAAGACAACCG |
| hicA.out.3' | TCGCTTCAGCGACAGTTTCG |
| lon.out.5' | TGCGTTCCATCGTAGAAGCC |
| lon.out.3' | GATCCGCCATCTAACTTAGC |
| recA.ext-5 | gtgctatcttgtccggcata |
| recA.ext-3 | cagaaaacgctggatcttaac |
| ccdB.ext-5 | tgtgttgtatctctggactgtg |
| ccdB.ext-3 | tcctgaccgttctgtccgtcac |
